# Cardiac Surgery in Cancer Patients: Clinical Dilemmas and Decision-Making Challenges

**DOI:** 10.3390/cancers18050748

**Published:** 2026-02-26

**Authors:** Kalliopi Keramida, Dorothea Tsekoura, Apostolοs Roubelakis, Helena Michalopoulou, Vasiliki Androutsopoulou

**Affiliations:** 1 General Anticancer Oncological Hospital, Agios Savvas, 11522 Athens, Greece; keramidakalliopi@hotmail.com; 2 2nd Department of Surgery, Aretaieio University Hospital, National and Kapodistrian University of Athens, 11528 Athens, Greece; dtsekoura@hotmail.com; 3 3rd Department of Cardiac Surgery, Athens Medical Center, 11526 Athens, Greece; roube@hotmail.com; 4 Department of Cardiology, Laiko General Hospital of Athens, 11527 Athens, Greece; helena.michal@gmail.com; 5 Department of Cardiothoracic Surgery, University Hospital of Larissa, 41110 Larissa, Greece

**Keywords:** cardio-oncology, cardiac surgery, cancer survivors, multidisciplinary decision-making, frailty, surgical risk stratification, transcatheter cardiac interventions, perioperative management

## Abstract

**Background:** Improvements in cancer detection and treatment have led to a growing population of cancer survivors who subsequently develop cardiovascular (CV) disease requiring cardiac surgery. **Methods:** This narrative review synthesizes contemporary evidence from observational studies, registries, meta-analyses, and international guidelines addressing cardiac surgery in patients with active or prior cancer. **Results:** Cancer patients undergoing cardiac surgery face heightened risks related to hypercoagulability, bleeding, immunosuppression, frailty, and prior exposure to chemotherapy or radiotherapy. Conventional surgical risk scores inadequately capture cancer-specific factors such as tumor biology, prognosis, treatment trajectory, and frailty, often underestimating operative risk. Timing of surgery must balance CV urgency against oncologic priorities, with urgent cardiac conditions necessitating prompt intervention irrespective of cancer status, while stable disease allows individualized sequencing. Transcatheter and minimally invasive approaches increasingly play a key role, particularly in patients with limited life expectancy, hostile thoracic anatomy, or high surgical risk. Outcomes are heterogeneous and largely driven by cancer stage and prognosis rather than cardiac pathology alone. **Conclusions:** Cardiac surgery can be lifesaving and restore eligibility for definitive oncologic therapy in carefully selected patients, particularly those with early-stage or potentially curable malignancies. In advanced cancer, the benefits of invasive intervention must be weighed against perioperative burden, quality of life impact, and overall prognosis. Multidisciplinary, patient-centered decision-making within specialized cardio-oncology teams is essential. Future priorities include the development of cancer-specific risk prediction models, prospective surgical registries, and greater integration of quality-of-life outcomes into clinical decision-making.

## Simple Summary

Advances in cancer therapy have improved survival, resulting in a growing number of patients who develop cardiovascular disease requiring surgical intervention. Cardiac surgery in patients with active or prior cancer presents complex clinical dilemmas, as clinicians must balance cardiac urgency, oncologic prognosis, treatment-related toxicity, frailty, and quality-of-life considerations. This review synthesizes current evidence on the epidemiology, perioperative risks, timing, and outcomes of cardiac surgery in oncology populations. We highlight the limitations of existing surgical risk scores, discuss the role of transcatheter and minimally invasive alternatives, and emphasize the central importance of multidisciplinary decision-making. An individualized, patient-centered approach is essential to ensure that cardiac interventions are both clinically meaningful and aligned with oncologic goals.

## 1. Introduction

The coexistence of cardiovascular disease (CVD) and cancer has emerged as one of the most complex challenges in contemporary clinical practice. Advances in cancer detection and therapy have significantly improved survival rates across a wide spectrum of malignancies, transforming many cancers into chronic conditions [1,2]. As a result, a growing proportion of patients live long enough to develop structural heart disease requiring invasive or surgical intervention. In parallel, cancer therapies—including chemotherapy, radiotherapy (RT), and targeted agents—contribute directly to coronary, valvular, pericardial, and myocardial pathology, reinforcing the bidirectional interaction between malignancy and cardiovascular disease [3,4].

Despite this expanding clinical overlap, patients with active cancer have historically been excluded from major cardiac surgical trials and remain underrepresented in conventional surgical risk models, creating substantial uncertainty in perioperative decision-making [5,6]. Consequently, clinicians must balance cardiac urgency, oncologic prognosis, treatment-related toxicity, frailty, and quality-of-life considerations in the absence of dedicated cancer-specific surgical frameworks.

According to the National Cancer Institute, “a cancer survivor” is any individual from the time of diagnosis through the remainder of life. This definition includes both individuals living with active malignancy and those who are disease-free following treatment. In contrast, “active cancer” is commonly defined in clinical and cardiovascular (CV) literature as a diagnosis within the preceding six months, ongoing or recent systemic therapy, or the presence of recurrent, regionally advanced, or metastatic disease [7]. This review addresses cardiac surgery considerations across the oncologic continuum, including patients with active cancer and those with a history of malignancy in remission. While these populations share certain vulnerabilities, the timing, perioperative risk profile, and ethical considerations differ substantially.

This review aims to provide an integrated and clinically applicable framework for cardiac surgical decision-making in patients with active or prior cancer. Key topics include evolving epidemiological trends, limitations of current surgical risk assessment models, cancer-specific perioperative considerations, comparative outcomes of surgical and transcatheter strategies, and ethical dimensions of care. As a narrative review, interpretative discussion is integrated within each thematic section to allow domain-specific synthesis of available evidence.

## 2. Epidemiology and Clinical Relevance

In contemporary cardiac surgery registries, the reported prevalence of patients with a prior history of malignancy generally ranges between approximately 1.9% and 4.2%; in contrast, separate cohorts specifically examining patients undergoing cardiac surgery in the setting of active cancer report proportions ranging from approximately 3.4% to 7%, reflecting differences in inclusion criteria and denominators across studies [5]. Several cardiac pathologies requiring surgery are more common among cancer patients. Coronary artery disease (CAD) is highly prevalent, driven by shared risk factors and accelerated by cancer therapies such as RT-induced coronary injury and chemotherapy-induced vascular toxicity [8]. RT-induced CAD typically presents years after thoracic irradiation and may involve ostial or proximal coronary segments, often necessitating surgical revascularization [9]. The prevalence of malignancy among patients undergoing coronary artery bypass grafting (CABG) has increased over time: 8.3% in the SWEDEHEART nationwide cohort (rising from 3.8% to 14.8%, 1997–2015) and from 7.0% to 12.6% in the U.S. (2003–2015) [10,11].

Valvular heart disease is another important surgical indication. Thoracic RT is strongly associated with progressive aortic and mitral valve fibrosis and calcification, with a long latent interval and increasing burden over time; observational data indicate actuarial rates of moderate-to-severe valve dysfunction of ~1% at 10 years, 4% at 15 years, and 6% at 20 years after mediastinal RT [12]. Consistent with this delayed phenotype, surgical series of patients undergoing first-time valve operations after prior RT report a mean interval of approximately ~19 years from RT to valve surgery, highlighting the need for long-term surveillance in cancer survivors [12,13].

Malignancy confers increased vulnerability to infective endocarditis (IE) through a combination of cancer-related and treatment-related factors. Immunosuppression, frequent invasive procedures, prolonged hospitalization, and the widespread use of indwelling central venous catheters or implantable ports increase the risk of bloodstream infection and facilitate endocardial seeding. In addition, pre-existing valvular abnormalities may provide a permissive substrate for infection once bacteremia occurs. Consistent with these, the prevalence of cancer among patients with IE ranges from approximately 5.6% to 17.6%, with one recent cohort reporting a prevalence of 11.6% [14]. Cohort data describe predominantly left-sided involvement, poor overall prognosis (median survival < 1 year in one cancer–IE cohort), and a substantial burden of *S. aureus* infection [15]. In parallel, non-bacterial thrombotic endocarditis (NBTE) is an uncommon but clinically consequential entity, with overall incidence estimates historically derived from autopsy series (≈0.9–1.6%) [16,17]. Among patients diagnosed premortem in contemporary clinical series, malignancy is one of the most frequent associated conditions (≈52.1%), and cancer-associated NBTE commonly involves left-sided valves with a high burden of embolic presentation and mortality [18]. In NBTE, anticoagulation and treatment of the underlying condition are central; the role of surgical intervention is limited, typically in the settings of heart failure, recurrent embolic events despite medical therapy, or rarely, for acute valve rupture [19].

Arrhythmias are common in cancer patients, particularly atrial fibrillation (AF), which is exacerbated by systemic inflammation, metabolic derangements, and chemotherapy [20,21]. Although most AF cases are treated medically or with catheter-based approaches, concomitant AF in patients already referred for open-heart surgery is clinically relevant: Society of Thoracic Surgeons (STS) data indicate that in 2022, ~43% of patients with documented AF undergoing first-time, nonemergent cardiac surgery received concomitant surgical ablation, underscoring the magnitude of the problem [22].

Pericardial disease is a clinically relevant complication in patients with cancer and cancer survivors, occurring both as a consequence of thoracic RT and as a manifestation of direct malignant involvement of the pericardium [23,24,25]. In contemporary cohorts of patients with locally advanced lung cancer treated with thoracic chemoradiation, the cumulative incidence of pericardial effusion exceeds 30% within the first year of treatment [26]. Pericardiocentesis is the predominant strategy; however, surgical pericardial window formation is required in a substantial minority (≈16%) and is associated with lower recurrence rates compared with percutaneous drainage [27]. Constrictive pericarditis, particularly following mediastinal RT, is a rare but severe late manifestation and often necessitates pericardiectomy, associated with higher operative risk and poorer long-term outcomes compared with other etiologies [28,29].

Finally, advanced heart failure is increasingly encountered in cancer survivors and may necessitate surgical or device-based strategies in the advanced stage. Registry-based and cohort analyses suggest that left ventricular assist device implantation is feasible in selected patients with active cancer or with a history of cancer, albeit with differences in bleeding risk and access to transplantation [30,31,32].

Thus, epidemiological evidence underscores the growing importance of cardiac surgery in oncology populations. This trend highlights the need for careful patient selection, multidisciplinary collaboration, and the development of cancer-specific risk prediction models that can better account for the interplay between CV and oncologic prognoses.

## 3. Special Considerations in Patients with Active Cancer

**Figure 1:**
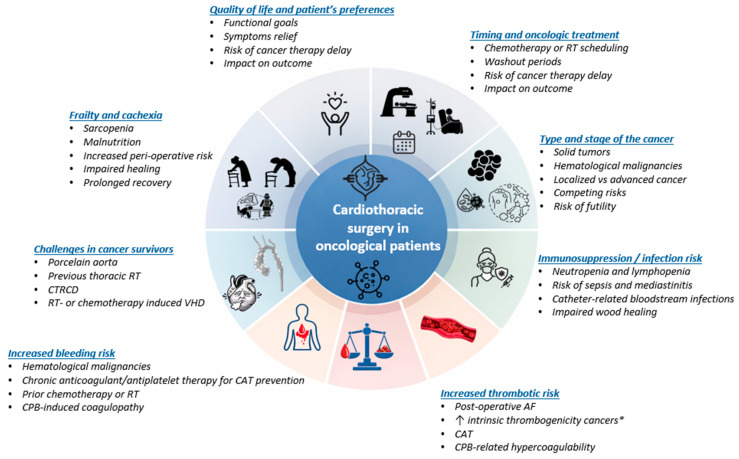
Cancer-specific determinants modifying cardiothoracic surgical risk. This schematic summarizes the principal oncologic modifiers influencing perioperative risk and outcome. These include hypercoagulability and thrombotic propensity, bleeding risk related to myelosuppression and anticoagulant exposure, frailty and cancer-associated cachexia, immunosuppression and infection vulnerability, and structural sequelae of prior radiotherapy or cardiotoxic therapies. These interrelated domains act synergistically and must be integrated into preoperative risk stratification within a multidisciplinary framework. CPB: cardiopulmonary bypass, CAT: cancer-associated thrombosis, RT: radiotherapy, VHD: vascular heart disease, CTRCD: cancer-therapy-associated cardiac dysfunction, AF: atrial fibrillation. * examples: pancreatic, gastric, lung, myeloproliferative malignancies.

Cardiac surgery in patients with active cancer entails a unique constellation of risks that extend beyond conventional perioperative considerations. Cancer and its treatments profoundly affect hemostasis, immune function, metabolic reserve, and physiological resilience, creating a fragile equilibrium in which thrombotic and hemorrhagic risks coexist, recovery is often delayed, and susceptibility to complications is heightened. Recognition of these cancer-specific factors is essential for accurate risk stratification, perioperative planning, and individualized decision-making. The major cancer-specific factors influencing perioperative risk, timing, and outcomes of cardiothoracic surgery are summarized in Figure 1.

### 3.1. Thrombotic Risk

Malignancy is a well-established prothrombotic condition and a leading cause of secondary venous and arterial thrombosis. Cancer-associated thrombosis (CAT) is driven by multiple mechanisms, including tumor-derived tissue factor expression, release of procoagulant microparticles, inflammatory cytokine-mediated endothelial activation, suppression of natural anticoagulant pathways, and impaired fibrinolysis. In addition, neutrophil extracellular traps (NETs) provide a structural scaffold for platelet adhesion and fibrin deposition, linking innate immunity with thrombogenesis [33,34,35,36].

In the context of cardiac surgery, this hypercoagulable milieu is further amplified by surgical trauma, cardiopulmonary bypass (CPB), systemic inflammation, endothelial injury, and postoperative immobility. Observational studies in general cardiac surgery populations report postoperative incidences of deep vein thrombosis of approximately 3.2%, pulmonary embolism of 0.6%, and fatal pulmonary embolism of 0.3%, with substantially higher rates observed in patients with active malignancy [37,38]. Thrombotic risk is particularly pronounced in cancers with high intrinsic thrombogenicity—such as pancreatic, gastric, lung, and brain tumors—as well as in patients receiving cisplatin-based chemotherapy, anti-angiogenic agents, or immunomodulatory therapies [39]. Myeloproliferative neoplasms further predispose patients to severe procoagulant states and, in advanced cases, disseminated intravascular coagulation [5]. Postoperative atrial fibrillation, which may occur in up to half of oncology patients undergoing cardiac surgery, further increases thromboembolic risk and often necessitates early initiation of anticoagulation, particularly in those undergoing valve surgery or with prior thrombotic events [5].

Early initiation of pharmacologic venous thromboembolism (VTE) prophylaxis, ideally within 24 h postoperatively, should be considered in patients at heightened procedural risk, including those undergoing open thoracoabdominal aortic aneurysm repair, thoracic endovascular aortic repair, infra-inguinal bypass graft surgery, carotid endarterectomy, or those with active malignancy [40]. Low molecular weight heparin (LMWH) is preferred over unfractionated heparin due to the increased risk of heparin-induced thrombocytopenia in cardiac and vascular surgery [40]. Early pharmacologic prophylaxis is associated with a significant reduction in postoperative pulmonary embolism (PE) (relative risk [RR] 0.45, 95% CI 0.28–0.71) and symptomatic VTE (RR 0.44, 95% CI 0.28–0.71), without a concomitant increase in clinically significant bleeding, including cardiac tamponade or the need for re-exploration [37]. Intermittent Pneumatic Compression (IPC) is a non-invasive mechanical prophylaxis method for VTE. It utilizes inflatable garments applied to the legs to enhance blood flow, thereby reducing the risk of deep vein thrombosis (DVT) and PE [41].

Arterial thrombotic complications, such as perioperative myocardial infarction, ischemic stroke, and graft thrombosis are of particular concern in patients with active cancer, reflecting the interplay of cancer-associated hypercoagulability, anticancer therapy-related acute coronary events and systemic inflammation. While randomized trials specifically addressing post-coronary artery bypass grafting (CABG) in cancer patients are scarce, current consensus guidelines emphasize aspirin as the cornerstone of antiplatelet therapy, recommending early reinitiation postoperatively to reduce graft occlusion and adverse ischemic events, with long-term continuation when bleeding risk is acceptable [42].

### 3.2. Bleeding Risk and Hemostatic Challenges

Bleeding remains one of the most frequent and clinically significant complications of cardiac surgery, particularly in procedures requiring CPB. CPB induces an acquired coagulopathy through hemodilution, platelet activation and dysfunction, consumption and dilution of coagulation factors, and activation of fibrinolysis following blood contact with artificial surfaces [43]. These hemostatic alterations increase perioperative blood loss and transfusion requirements, which are independently associated with adverse outcomes, including prolonged intensive care unit stay and increased mortality. Importantly, re-exploration for bleeding after cardiac surgery carries a significantly increased risk of postoperative mortality and morbidity [44].

Patients with cancer are at particularly high risk of hemorrhagic complications. Hematologic malignancies are associated with thrombocytopenia, qualitative platelet dysfunction, anemia, impaired coagulation factor synthesis, and endothelial injury resulting from prior chemotherapy and RT [45]. In addition, many oncology patients undergoing cardiac surgery receive chronic anticoagulant or antiplatelet therapy for primary or secondary prevention of CAT, further narrowing the therapeutic window between thrombosis and bleeding.

Preoperative assessment should focus on identifying anticoagulant exposure, timing of last dose, renal function, platelet count, and prior bleeding history. Intraoperatively, conventional coagulation assays are often insufficient for real-time decision-making. Point-of-care viscoelastic testing enables rapid, global assessment of clot initiation, strength, propagation, and fibrinolysis, facilitating goal-directed hemostatic management and reducing unnecessary transfusion [46]. Antifibrinolytic therapy, particularly tranexamic acid, has consistently demonstrated efficacy in reducing perioperative blood loss without an increase in mortality or thrombotic complications and should be considered standard adjunctive therapy in this high-risk population [47].

### 3.3. Frailty and Cachexia

Frailty is a multidimensional syndrome characterized by diminished physiological reserve and impaired tolerance to stressors and is highly prevalent among older adults with cancer [48,49]. In advanced cancer, frailty is even more common and strongly associated with all-cause mortality [50]. Although cancer-specific data in cardiac surgery populations remain limited, extensive evidence from cardiac surgical cohorts demonstrates that frailty and pre-frailty are independently associated with increased operative mortality and reduced mid-term survival [51]. Frail patients typically exhibit hypoalbuminemia, higher comorbidity burden, and impaired functional status—features that overlap substantially with cancer-related systemic decline.

In clinical practice, frailty should be assessed using validated and reproducible instruments rather than subjective clinical impression alone. Commonly applied tools include the Fried Frailty Phenotype, which evaluates weight loss, exhaustion, grip strength, gait speed, and physical activity, and the Clinical Frailty Scale, a rapid global assessment of functional status that has demonstrated prognostic value in cardiac surgical populations [52]. The cumulative deficit-based Frailty Index proposed by Rockwood and colleagues may also be used when a more multidimensional assessment is desired [53,54]. In oncology populations, frailty has been consistently associated with increased postoperative morbidity and mortality, supporting its systematic incorporation into preoperative evaluation.

Cachexia represents a distinct but closely related metabolic syndrome defined by progressive skeletal muscle loss and systemic inflammation. Cachexia frequently coexists with frailty and is highly prevalent in advanced malignancy and in gastrointestinal and lung cancers, where catabolic pathways erode skeletal and cardiac muscle mass, resulting in increased operative mortality and prolonged hospitalization [22]. Cachexia contributes to mitochondrial dysfunction, neurohormonal activation, and myocardial atrophy, processes encompassed within the concept of cancer-related cardiac wasting, which adversely affects ventricular performance and perioperative resilience [55]. The prevalence of cachexia ranges from 5 to 20% in chronic heart failure to as high as 60–80% in advanced cancer, underscoring its clinical relevance at the intersection of oncology and CVD.

Cachexia and nutritional vulnerability should likewise be formally assessed. Screening tools such as the Mini Nutritional Assessment (MNA), evaluation of unintentional weight loss and serum albumin levels, and imaging-based assessment of sarcopenia—particularly CT-derived skeletal muscle index in cancer patients—have demonstrated prognostic significance in oncologic cohorts [56,57].

Importantly, conventional cardiac surgery risk models, including EuroSCORE II and the Society of Thoracic Surgeons (STS) score, inadequately account for frailty and cancer-related vulnerability, despite formal inclusion of malignancy in the STS score [58,59]. As cancer survival improves and the number of patients presenting with concomitant malignancy and advanced CVD increases, systematic integration of frailty and cachexia assessment into preoperative evaluation becomes essential to balance surgical risk, oncologic prognosis, and anticipated quality of life (QoL) benefit [5].

### 3.4. Immunosuppression and Risk of Infection

Immunosuppression represents a critical determinant of perioperative outcomes in cancer patients undergoing cardiac surgery. Both malignancy and its treatments—including cytotoxic chemotherapy, targeted therapies, RT, immune checkpoint inhibitors, hematopoietic stem cell transplantation, and prolonged corticosteroid exposure—disrupt both innate and adaptive immune responses, impair wound healing, and compromise host defense mechanisms. These mechanisms increase susceptibility to infection and poor postoperative outcomes [23,60]. In the context of cardiac surgery—where large surgical fields, prolonged operative times, implantation of foreign material, and CPB-induced systemic inflammation are common—immunosuppressed patients face a substantially increased risk of postoperative infections, including surgical-site infection, mediastinitis, pneumonia, bloodstream infection, and sepsis. CPB itself has been shown to cause immune dysfunction via quantitative and qualitative alterations in leukocyte subpopulations and T-cell function, and may induce a transient immunosuppressed state that contributes to infection risk [61].

Neutropenia and lymphopenia, which are common in patients with hematologic malignancies or as a consequence of myelosuppressive therapies, further amplify the risk of infection by reducing the primary cellular defenses against pathogens. Impairment of mucosal barriers, presence of indwelling central venous catheters, and metabolic derangements (e.g., malnutrition, anemia) add to this vulnerability. In addition, opportunistic pathogens—organisms that ordinarily cause limited disease in immunocompetent hosts—cause more severe and difficult-to-treat infections in immunosuppressed patients. Importantly, postoperative infectious complications following cardiac surgery are associated with high morbidity and mortality. In contemporary cardiac surgical cohorts, postoperative infection rates—especially pneumonia and SSIs—remain at a clinically significant level and have been linked to prolonged intensive care unit stay, increased healthcare resource utilization, and delayed recovery. These complications not only increase short-term mortality but can also delay the continuation of oncologic therapy, adversely affecting overall cancer outcomes [62].

Preoperative optimization should therefore include careful timing of surgery relative to chemotherapy or immunotherapy cycles and thorough assessment of immune status (including evaluation of leukocyte counts and functional status). Implementation of aggressive infection-prevention strategies is essential; these include appropriate perioperative antimicrobial prophylaxis based on current guidelines (administered within 60 min before incision and tailored to the patient’s risk profile), strict perioperative glycemic and nutritional control, minimization of invasive devices such as urinary catheters and central venous lines, and early postoperative mobilization [63]. In selected high-risk patients, multidisciplinary discussion involving oncology, infectious disease, anesthesiology, and cardiac surgery teams is essential to balance surgical urgency against immunologic recovery potential and to mitigate the risk of severe postoperative infection. A proactive, team-based approach allows for individualized perioperative planning, optimization of organ function, and anticipation of complications in order to improve both short-term surgical outcomes and long-term cancer-related outcomes [60].

## 4. Special Consideration in Patients with History of Cancer

In cancer survivors who have not developed overt myocardial dysfunction or heart failure from prior cardiotoxic therapy, the dominant issue for cardiac surgery is often RT-induced anatomic changes. Prior mediastinal RT can produce a complex pattern of coronary, valvular and pericardial disease together with dense mediastinal fibrosis, adhesions and calcification of the ascending aorta (“porcelain aorta”), all of which increase surgical difficulty and perioperative risk [64]. These changes may complicate re-entry and cannulation, increase bleeding and stroke risk when clamping a heavily calcified aorta, and limit the use of the internal mammary arteries as conduits because of RT damage or impaired sternal perfusion [65]. Experience from single-center cohorts and small observational studies of cardiac surgery after mediastinal RT indicates higher perioperative and long-term risk, underscoring the importance of meticulous pre-operative multimodality imaging (echocardiography, CT of the chest and aorta, and coronary imaging) to delineate the extent of RT-associated heart disease and plan the operative strategy [66]. The 2022 ESC cardio-oncology guidelines and expert reviews therefore recommend that, in such survivors, pre-existing RT injury to the mediastinum and great vessels is treated as a major determinant of risk and strategy—often prompting consideration of alternative cannulation sites, off-pump or hybrid procedures, or transcatheter valve and coronary interventions when anatomy and cancer prognosis allow [3].

## 5. Risk Stratification, Timing of Surgery and Decision-Making

Risk scores and scoring systems for assessing surgical risk in cancer patients undergoing cardiac surgeries primarily rely on general cardiac surgery risk models, with some inclusion of cancer-related factors. Two widely used risk scores in cardiac surgery are the EuroSCORE II [67] and the STS score [68]. While both scores are validated tools for predicting operative mortality and morbidity, only the STS score incorporates specific cancer-related variables such as whether a patient has active cancer, a history of cancer within five years, prior mediastinal RT, or immunosuppression. However, the STS score does not account for the type or stage of cancer or for cancer-specific factors such as hypercoagulability, cachexia, prior RT, chemotherapy-induced cardiotoxicity limiting its specificity in oncology patients. Consequently, it is likely to underestimate surgical risk in oncology patients.

Currently, no dedicated risk scores exclusively tailored to the cardiac surgical population with detailed cancer characteristics exist, and preoperative assessment usually relies on these general tools. The lack of cancer-specific granularity highlights the need for further development of risk stratification tools that integrate both oncologic and CV prognosticators. Oncologic parameters such as cancer type, TNM stage, time since diagnosis, and anticipated survival are critical. For example, patients with early-stage breast or colorectal cancer, whose 5-year survival exceeds 70%, may benefit from aggressive cardiac surgery that restores candidacy for curative oncologic treatment.

In the context of cardiac surgery for cancer survivors or patients with a history of malignancy, comprehensive risk stratification must extend beyond traditional cardiac scores to include frailty assessment. Pioneering work by Afilalo et al. demonstrated that adding frailty and disability metrics to standard cardiac-surgery risk models markedly improves identification of elderly patients at high risk of mortality and major morbidity [69]. Kim et al. further validated this in CABG patients, showing that higher frailty index scores correlate independently with more postoperative complications, prolonged hospitalization, and worse survival [70]. Importantly, in the oncologic setting, a systematic review by Shaw et al. found that frailty among elective cancer-surgery patients was associated with adjusted odds ratios of ~3.0 for 30-day mortality and ~2.4 for major complications [71], while Handforth et al. reported a strong association between frailty and poorer long-term survival in cancer cohorts [49]. Together, these findings underscore that in cancer survivors undergoing cardiac surgery, frailty reflects diminished physiological reserve from cumulative cancer treatment, sarcopenia, malnutrition, and functional decline. As such, frailty emerges as a critical determinant of procedural risk, recovery potential, and anticipated QoL benefit. Routine incorporation of frailty measures into the pre-operative work-up, combined with targeted prehabilitation and shared decision-making, therefore becomes essential to tailor the therapeutic strategy and align it with the patient’s long-term oncologic prognosis and life goals.

## 6. Timing of the Surgery

The timing of cardiac surgery in patients with cancer is one of the most complex aspects of decision-making and requires individualized assessment of urgency, oncologic trajectory, and comorbidity burden. In acute life-threatening scenarios, such as left main coronary occlusion, infective endocarditis with hemodynamic compromise or severe aortic stenosis with cardiogenic shock, superior vena cava syndrome, acute aortic dissection or acute mitral valve regurgitation, surgery cannot be delayed irrespective of cancer status. In these cases, short-term survival depends on timely cardiac intervention, and oncological considerations become secondary.

In contrast, in clinically stable patients with non-critical disease, surgery may often be deferred until the oncologic plan is clarified and perioperative risks are optimized. The interaction between cardiac surgery and cancer therapy is particularly relevant in this context. Surgery performed during active chemotherapy is associated with increased risks of myelosuppression, bleeding, and infection, whereas prior chest RT complicates cardiac surgery through fibrosis, adhesions, and mediastinal scarring. Moreover, both chemotherapy and RT may exert direct or indirect CV toxicity, supporting the rationale for optimizing cardiac function before oncologic treatment when feasible. Conversely, when cancer therapy is urgent and cardiac disease is stable, cancer treatment may proceed first, accompanied by close CV surveillance and management.

When both cardiac and oncologic surgical interventions are required, the optimal sequencing remains controversial and must be individualized. As a general principle, severe cardiac disease that threatens perioperative safety should be addressed first in order to stabilize hemodynamics and reduce the risk of subsequent cancer surgery. This approach is particularly favored in patients requiring complex cardiac procedures or presenting with hemodynamic instability, in whom lung or other solid-organ resections are often postponed for approximately two months following cardiac surgery [5].

Small cohorts report acceptable outcomes when tumor surgery is staged ~1–3 months after CABG (no in-hospital deaths/CV events in one series), but these are limited and not prescriptive [72]. Importantly, a systematic review by Hanna et al. [73] spanning >2500 publications demonstrated that for several solid tumors—including head and neck, bladder, colon, and lung cancers—each four-week delay in time to definitive cancer treatment is associated with a 6–8% increase in mortality, underscoring the need to balance surgical recovery with oncologic urgency.

When feasible, subsequent cancer surgery is advised to be delayed to ~3 months (≈100 days) after cardiac surgery to reduce perioperative risk [74]. However, in urgent oncologic scenarios, staged surgery within 1–3 months—or in selected cases, simultaneous procedures—may be appropriate following multidisciplinary team (MDT) evaluation. A single-stage surgical approach may be considered in selected patients with concomitant cardiac disease and resectable lung or kidney cancer [75,76,77,78]. Simultaneous cardiac and oncologic surgery offers the benefit of the advantage of a single anesthetic exposure and avoidance of treatment delays. Nevertheless, combined procedures increase operative time, complexity, and perioperative stress and may be associated with higher blood loss. Meta-analyses report acceptable perioperative mortality rates (<5%) for combined heart and lung resections, particularly when Off-Pump Coronary Artery Bypass (OPCAB) techniques are employed [79,80]. Careful patient selection is essential, favoring individuals with limited, resectable primary tumors, absent or minimal metastatic disease, adequate cardiopulmonary reserve, and a realistic expectation of oncologic benefit. Institutional experience with combined procedures is critical.

The timing of surgery relative to systemic anticancer therapy requires particular attention. Cytotoxic chemotherapy is generally avoided in the immediate perioperative period, and surgery is typically staged between treatment cycles once neutrophil and platelet counts have recovered. After neoadjuvant therapy, many pathways target an interval of approximately four–eight weeks before surgery to allow recovery from marrow suppression and systemic toxicity [80,81]. Certain agents necessitate specific washout periods; for example, bevacizumab is commonly withheld for six to eight weeks before and at least four to eight weeks after major surgery, with regulatory guidance recommending a minimum of 28 days before and after surgery and confirmation of adequate wound healing [82,83]. The optimal timing of surgery following immune checkpoint inhibitor (ICI) therapy is less well defined; expert consensus in early-stage non-small cell lung cancer supports surgery approximately four to six weeks after the last neoadjuvant dose, whereas other guidance suggests that ICIs may be continued peri-operatively with appropriate monitoring [80]. Prior mediastinal/thoracic RT is linked to higher perioperative risk and worse outcomes after cardiac surgery, informing pre-operative planning and surveillance rather than mandating a fixed delay [84,85]. Postoperatively, adjuvant systemic therapy is commonly re-initiated within a few weeks once recovery permits, as prolonged delays beyond this window are associated with worse oncologic outcomes.

## 7. Decision-Making Framework and Patient Selection

Decision-making regarding cardiac surgery in patients with current or prior cancer should be undertaken within a formal MDT that includes medical, hematologic, and radiation oncology; cardiology; cardiac surgery; cardiac anesthesia and intensive care; and, when appropriate, geriatric and palliative care specialists [86]. The goal of this collaborative approach is to balance tumor biology and anticipated oncologic benefit against the urgency of the CV condition, perioperative CV and hematologic risk, and patient values and preferences [86,87,88]. The 2022 ESC Cardio-Oncology Guidelines explicitly recommend MDT-based management within specialized cardio-oncology services for patients at risk of cancer therapy-related CV toxicity, principles that extend naturally to surgical decision-making where interruption or sequencing of cancer therapy must be carefully weighed [3]. ESMO consensus recommendations similarly emphasize coordinated, multidisciplinary cardio-oncology care throughout the cancer continuum [89]. A practical stepwise decision-making framework is illustrated in the Graphical Abstract.

From the CV perspective, the contemporary Heart Team model—now a Class I recommendation in revascularization guidelines—provides an operational framework for shared decision-making, standardized workflows, and documented accountability. Cardio-oncology-specific adaptations of this model explicitly integrate oncology and advanced imaging expertise [90]. In the absence of cancer-specific surgical risk scores, MDT deliberation is particularly important to individualize the timing of surgery relative to chemotherapy or RT, determine antithrombotic strategies, and select the most appropriate surgical approach, especially in scenarios where active malignancy complicates valve or coronary interventions [5]. Perioperative consensus statements for cardiac surgery, including enhanced recovery after surgery (ERAS) pathways, further underscore the importance of multidisciplinary coordination in prehabilitation, anesthesia, and postoperative care—elements that can be tailored to oncology-specific needs through MDT planning [91].

Oncologic stage and prognosis are central to whether and when to proceed with cardiac surgery [3,92]. Patients with early-stage, potentially curable disease are the most likely to gain net benefit from aggressive cardiac interventions that enable timely, definitive cancer therapy. For example, in localized colon cancer—where curative surgery is standard—prompt treatment of a cardiac condition that would otherwise preclude oncologic surgery is often justified [93]. In contrast, malignancies such as pancreatic adenocarcinoma or advanced lung cancer continue to carry poor survival despite contemporary therapies, and invasive cardiac surgery in these settings is less likely to change overall prognosis, necessitating careful weighing of competing risks [94].

Hematologic malignancies (HM) pose distinct perioperative hazards because of disease- and therapy-related cytopenias, immunosuppression, bleeding diathesis, and heightened infection risk, all of which mandate tailored perioperative planning [95]. High-quality evidence defining exact intervals is limited; thus, decisions are individualized within a MDT, with a pragmatic approach favoring surgery between chemotherapy cycles once blood counts have recovered and infectious risk is controlled, and neutrophil and platelet counts have recovered and infectious risk is controlled [96]. Concerns that CPB may accelerate malignant progression should not, in isolation, delay necessary surgery, as a best-evidence analysis found no increase in long-term mortality or cancer progression in hematologic malignancy [96]. Contemporary series suggest that when patients are carefully selected and optimized, early postoperative outcomes are broadly similar to matched controls, supporting timely surgery when cardiac indications are urgent [97]. In chronic lymphocytic leukemia (CLL) cohorts, in-hospital mortality is generally comparable to non-CLL patients, but transfusion needs and nonelective readmissions are higher, arguing to operate in a nadir-free window and to deploy blood-conservation strategies [98,99].

System-level factors also influence outcomes. Data indicate that centers with greater experience managing patients with hematologic malignancies undergoing cardiac surgery achieve lower rates of acute kidney injury and respiratory failure, supporting referral to experienced centers for time-sensitive or complex cases [100]. Disease- and therapy-specific considerations (e.g., valve intervention choices in CML receiving BCR-ABL tyrosine-kinase inhibitors) may further influence both timing and surgical strategy, reinforcing the value of close coordination between the Heart Team and hematology [101]. Ultimately, long-term outcomes depend on durable hematologic remission and immunologic stability [95,102], while active, uncontrolled hematologic disease and severe cytopenias predict poorer results.

## 8. Surgical Considerations and Outcomes

### 8.1. Type of Surgery and Modality Selection

Management of ischemic heart disease in active or advanced cancer should be multidisciplinary and patient-centered, explicitly incorporating cancer type and biology, stage and symptoms, comorbidities, life expectancy, and the feasibility/timing of CABG versus percutaneous coronary intervention (PCI) [3]. In ST-elevation myocardial infarction (STEMI) and most non-ST-elevation myocardial infarction (NSTEMI) with active cancer, PCI is recommended per general acute coronary syndrome guidance adapted to cardio-oncology (with careful bleeding risk mitigation) [103]. Cancer-related thrombocytopenia increases bleeding after PCI; radial access and abbreviated double antiplatelet therapy (DAPT) are commonly employed in high-bleeding-risk scenarios, with platelet-count-aware strategies endorsed by expert consensus and hematology guidance [103,104]. For stable CAD when 3-month DAPT is unsafe, some reviews allow 1-month DAPT with contemporary DES in selected high-bleeding-risk patients, but this must be individualized [105]. Conversely, cancer is also a prothrombotic state and observational data show higher stent-thrombosis and bleeding rates after PCI than in non-cancer comparators, underscoring the need to tailor antithrombotic intensity and duration [106].

The choice of revascularization modality in a patient with cancer should balance procedural risk, anticipated survival, and the oncology plan within an MDT framework [3,107]. In patients with limited life expectancy or ongoing cytotoxic therapy, PCI is often favored because it is less invasive than sternotomy/CABG and can minimize interruptions of anticancer treatment [103]. By contrast, for left main disease and many cases of complex multivessel CAD, CABG remains the standard of care to improve survival, with PCI reserved for anatomies where equivalent revascularization is achievable [108].

Prior thoracic RT commonly produces ostial/proximal coronary lesions and a “hostile chest,” making strategy selection nuanced; evidence is limited and decisions should be individualized by the MDT given surgical challenges and the feasibility of PCI [109]. Contemporary series suggest that CABG outcomes in patients with cancer are generally acceptable when the cancer prognosis is favorable, although perioperative bleeding and some complications are higher than in non-cancer cohorts [11,110]. In comparative terms, while PCI offers the advantage of reduced procedural invasiveness and shorter recovery time—particularly attractive in patients with limited life expectancy or ongoing oncologic therapy—CABG may provide superior long-term durability in anatomically complex disease when cancer prognosis is favorable. Thus, modality selection should balance expected oncologic survival against the need for durable coronary revascularization.

Valvular heart disease, especially AS after mediastinal RT, poses unique anatomic and perioperative hazards that favor less invasive strategies and careful MDT selection [111]. Transcatheter aortic valve replacement (TAVR) is an attractive alternative to surgical AVR (SAVR) in patients with frailty, hostile thoracic anatomy (e.g., porcelain aorta or prior chest RT), or active malignancy, consistent with contemporary ACC/AHA and ESC/EACTS guideline frameworks emphasizing age, life expectancy, anatomy, and frailty in modality choice [112,113]. Direct comparisons in oncology populations suggest that early outcomes following TAVR are generally comparable to SAVR in appropriately selected patients, with lower transfusion requirements and shorter hospitalization favoring transcatheter approaches in frail or actively treated cancer patients. However, longer-term survival differences appear largely driven by underlying malignancy rather than procedural modality, underscoring the importance of individualized decision-making based on oncologic trajectory.

In particular, prior chest RT and porcelain aorta are scenarios where observational data and expert pathways support preferring TAVR over SAVR due to higher surgical risk and embolic concerns with sternotomy and aortic clamping [114,115,116]. Decisions should explicitly consider life expectancy, as guidelines advise against valve intervention when expected survival is <1 year or QoL gain is unlikely [112,117].

Compared with SAVR, TAVR is less invasive, typically yields shorter length of stay, and in large databases, is associated with lower early mortality and bleeding (offset by higher pacemaker/stroke in some cohorts), factors that can minimize interruptions to cancer therapy [118]. After TAVR, non-cardiac operations (including oncologic procedures) have been performed safely within 30–90 days with no increase in major adverse events versus later timing, enabling earlier continuation of cancer care; by contrast, non-cardiac surgery after open cardiac surgery carries a lower risk when delayed to ~100 days [74,119,120].

For oncology status, multiple registries and meta-analyses show similar in-hospital/30-day outcomes for TAVR in cancer vs. non-cancer patients, whereas 1-year mortality is higher in those with active cancer—driven largely by malignancy rather than valve complications [121,122,123]. In survivors with prior thoracic RT, pooled analyses report comparable short-term TAVR safety to non-irradiated patients, supporting TAVR as a reasonable option when anatomy permits [124]. Overall, available evidence indicates that while transcatheter approaches may reduce early procedural burden, especially in RT-associated or high-risk patients, the dominant determinant of long-term outcome remains cancer stage and systemic disease progression rather than the choice between surgical and transcatheter intervention.

When transcatheter therapy is unsuitable (e.g., unfavorable annulus/root anatomy or need for concomitant surgical procedures), minimally invasive aortic valve replacement (MIAVR) via mini-sternotomy or right anterior mini thoracotomy offers less trauma, fewer transfusions, shorter ICU/hospital stay, and lower wound infection than full sternotomy in observational series, though large, randomized trials are lacking [125]. The infection risk rate is lower than that of median sternotomy [126]. These techniques seem to provide advantages for patients with malignancies; however, there is currently insufficient data from large randomized controlled trials.

Cancer-related pericardial effusion presenting with hemodynamic compromise or tamponade is a medical emergency that requires urgent drainage—typically echo-guided pericardiocentesis with extended catheter drainage, or surgical pericardial window when indicated [127]. Pericardiocentesis provides rapid symptom relief, but recurrence is common; extended catheter drainage (≈3–5 days) reduces recurrence to ~10%, yet re-accumulation remains higher than after surgical window/pericardiotomy [128]. Among surgical options, subxiphoid and thoracotomy/video-assisted thoracoscopic surgery (VATS) windows offer durable control; comparative series show lower effusion recurrence with thoracotomy/VATS at the cost of more postoperative pain/ventilatory time, whereas subxiphoid enables faster recovery but has higher recurrence [129,130,131]. Contemporary oncology cohorts also report that the pericardial window is safe and effective for tamponade control and palliation in cancer patients [132,133]. The choice of approach should weigh anticipated survival, likelihood of recurrence (e.g., positive cytology/tumor biology), and patient preferences within a cardio-oncology MDT [127].

Constrictive pericarditis—often related to prior thoracic RT or surgery—is probably under-recognized in oncology; pericardiectomy is the definitive therapy [127,134]. Perioperative mortality after pericardiectomy is ~5–10% in modern series and is higher with RT-associated constriction, with long-term outcomes strongly influenced by etiology [135,136,137]. In post-RT constriction specifically, studies consistently show worse early and late survival, underscoring the need for careful selection and management at experienced centers [136].

In malignant superior vena cava (SVC) syndrome, first-line management is endovascular stenting to achieve rapid symptom relief, partnered with disease-directed therapy (chemotherapy and/or RT) according to tumor histology [138,139]. Stenting typically provides improvement within hours to days and is now considered standard initial therapy in symptomatic patients, including those with recurrent or refractory symptoms [140,141]. Contemporary systematic reviews and meta-analyses report high technical success and favorable patency/recurrence profiles of endovascular therapy in malignant SVC obstruction [142]. Importantly, stenting does not treat the underlying malignancy, so oncologic therapy should proceed in parallel once venous decompression is achieved [138]. Surgical intervention (bypass/reconstruction) is rarely recommended for malignant SVC syndrome and is generally reserved for select cases (e.g., en bloc resection during curative thoracic tumor surgery) because outcomes in palliative malignant settings are poor and life expectancy is often limited [143,144]. Historical series of SVC bypass for malignant disease document limited survival despite symptom control, underscoring why endovascular palliation is preferred in most cancer patients [145].

### 8.2. Off-Pump vs. On-Pump Cardiac Surgery

Traditional on-pump cardiac surgery (i.e., with the use of a CPB circuit) can be performed in cancer patients. The use of CPB, however, is linked to a profound systemic inflammatory response and transient immunosuppression, characterized by cytokine release, complement activation, lymphocyte and natural killer (NK) cell dysfunction, coagulopathy and endothelial activation [61]. The above outcomes raise theoretical concerns for infection and possible influence on tumor biology. Nevertheless, on-pump cardiac surgery is mandatory for the surgical treatment of all cardiac pathologies (aorta, valves, coronary operations), including complex and concomitant cardiac disease.

Off-pump cardiac surgery can be performed in selected patients suffering from CAD. Off-pump CABG (OPCAB) is linked to reduced inflammatory and immunosuppressive response compared to on-pump cardiac surgery [61]. Therefore, OPCABG, where possible, is increasingly considered in cancer patients. Studies report comparable early mortality between OPCAB and on-pump CABG, with OPCAB showing reduced transfusion needs and fewer pulmonary complications, potentially relevant in oncologic patients who are immunocompromised. Long-term survival, however, depends largely on tumor burden and stage rather than CPB exposure; hence, the choice must remain individualized to cardiac anatomy, disease complexity, cancer stage and oncological plan [146,147].

The relationship between CPB and malignancy progression remains a topic of ongoing debate. Immune alterations could potentially reduce tumor immune surveillance and facilitate the dissemination or implantation of circulating tumor cells. Additionally, CPB-associated endothelial activation and shear stress may promote the adhesion of malignant cells to vascular structures, providing a biological rationale for metastatic spread [96]. Despite these concerns, clinical evidence to date does not support a significant oncologic disadvantage associated with CPB use. In a large retrospective cohort study, Pinto et al. found that patients undergoing CPB had only a modest and statistically non-significant association with subsequent cancer progression or mortality compared with non-CPB patients [148]. Similarly, Plumereau et al. examined 59 patients with hematologic malignancies who required CPB and observed no significant increase in early postoperative mortality or disease relapse; CPB was not an independent predictor of malignancy progression [149]. Experimental and translational studies continue to suggest possible mechanisms for tumor cell dissemination, yet these remain largely theoretical and unproven in clinical cohorts [96]. The majority of available evidence indicates that tumor biology and disease stage are the dominant determinants of postoperative recurrence and survival, rather than the use of extracorporeal circulation.

### 8.3. Peri and Postoperative Considerations

Perioperative complications are frequent. Cancer patients undergoing cardiac surgery have increased risks of bleeding, thromboembolic events, and postoperative infections. Prior chest RT complicates operations by inducing mediastinal fibrosis, impairing vascular conduit availability, and increasing the likelihood of wound complications and extensive aortic calcification (e.g., porcelain aorta) [8]. Excessive calcification of the ascending aorta can hinder aortic cannulation and clamping, potentially requiring alternative methods like peripheral arterial cannulation (e.g., femoral, axillary) or off-pump cardiac revascularization surgery. Additionally, subclavian artery stenosis or internal mammary fibrosis might limit its suitability for use as a graft option in CABG [5].

Due to the frailty of cancer patients and their exposure to chemotherapy and RT, there is an increased risk associated with cardiac surgery procedures and a higher likelihood of postoperative complications. Patients with current or past malignancies undergoing cardiac surgery face higher postoperative complications, including pulmonary embolism and venous thromboembolism, due to their typical hypercoagulable state. Haemorrhagic complications can also occur, particularly in patients with hematological malignancies. The high rate of transfusion could contribute to an increased incidence of respiratory complications and prolonged mechanical ventilation. Patients with malignancies also experience higher rates of sepsis and arrhythmias. On the other hand, cancer patients undergoing cardiac surgery have a similar short-term mortality rate to the general population [150].

### 8.4. Short- and Long-Term Surgical Outcomes

Survival outcomes are heterogeneous. Several series suggest that early postoperative mortality is higher in cancer patients, particularly those with active malignancy. However, among patients with early-stage cancers and curative oncologic treatment options, long-term survival after cardiac surgery may approximate that of patients without cancer. Conversely, patients with advanced metastatic disease derive little survival benefit, raising concerns of futility. Importantly, cardiac surgery can restore eligibility for potentially life-prolonging cancer therapies, underscoring its role in the continuum of multidisciplinary cancer care.

Carrascal et al. demonstrated that postoperative survival in cancer patients is closely associated with preoperative left ventricular function, the presence of chronic pulmonary disease, and the interval between cancer diagnosis and surgery [151]. Mistiaen et al. found that having a malignant tumor is the main predictor of 5-year survival after cardiac surgery [152]. They also discovered that nearly 50% of deaths were due to the progression of the primary malignancy, especially when the interval between cancer diagnosis and cardiac surgery was short. Other prognostic factors are chronic obstructive pulmonary disease, older age and decreased left ventricular function.

## 9. Surgery for Cardiac Tumors

Primary malignant cardiac tumors are rare. The most common are sarcomas, with angiosarcomas being the most frequent. Surgical resection offers the only potential for prolonged survival. Complete (R0) resection of limited disease, when possible, improves survival compared to partial resection or biopsy but is often not achievable [153,154]. If complete tumor resection is not possible, debulking may be considered to enable other, more effective modes of treatment. Newer registry and genomic studies confirm that multimodal therapy (surgery + adjuvant therapy) extends survival, though recurrence rates remain high [154,155].

Metastatic cardiac involvement usually occurs via hematogenous spread, lymphatic extension, direct invasion (lung, breast, mediastinal tumors), or IVC extension (renal cell carcinoma). It usually reflects advanced systemic disease. Surgery for these patients is usually for symptomatic relief or palliation, as metastatic tumors tend to cause obstruction, may trigger arrhythmias, and may cause thromboembolic events. Rarely, surgery offers a therapeutic benefit, especially in solitary, localized metastatic masses where the primary disease is controlled and a curative intent is considered. Outcomes depend on many factors, but mainly on the oncologic burden. Cardiac surgery for these patients usually offers symptomatic relief and may improve survival [99,156]. All planned cardiac surgery for primary or metastatic masses needs careful planning and MDT input, ideally in specialized centers [99].

## 10. Surgery for Cardiac Carcinoid Disease

Cardiac involvement in neuroendocrine tumors (carcinoid heart disease, CaHD) results from chronic exposure of the endocardium—particularly the right-sided heart—to vasoactive substances, leading to progressive fibrotic destruction of the tricuspid and pulmonary valves and ultimately right-sided heart failure [157]. Unlike primary or metastatic cardiac tumors, surgery in CaHD is not performed for tumor resection but almost exclusively for valve replacement, as multi-valvular involvement is common: up to 70–80% of patients require both tricuspid and pulmonary valve replacement because of diffuse plaque-like fibrosis affecting the entire right-sided outflow tract [158,159]. Surgical exposure is often challenging due to marked thickening and retraction of valvular and subvalvular structures and fibrosis extending into the right atrium and right ventricular outflow tract (RVOT), complicating prosthesis seating and annular mobilization [160]. Because CaHD patients frequently present severe right-heart dilation and progressively reduced RV reserve, outcomes are significantly better when surgery is performed before advanced or irreversible RV dysfunction develops [158,159]. Prosthesis choice is also specific: contemporary guidelines and consensus documents consistently recommend bioprosthetic valves in both tricuspid and pulmonary positions, given the high thrombosis risk of mechanical valves in the low-pressure right-sided circulation and the frequent need for systemic therapies that increase bleeding risk [158,161]. Perioperative management is specialized, requiring high-dose octreotide prophylaxis and continuous intraoperative infusion to prevent carcinoid crisis, which can manifest as life-threatening hypotension, bronchospasm, and arrhythmias [158,161]. Decisions regarding timing also incorporate the NET trajectory: surgery is generally offered when extracardiac neuroendocrine disease is controlled or controllable and expected survival is ≥12 months, ensuring that operative risk aligns with meaningful survival and functional benefit [3,161]. Because CaHD surgery involves multi-valve replacement, complex perioperative endocrine management, and coordination with liver-directed or systemic NET therapy, all major guidelines and expert series stress that these operations should be performed only in experienced, high-volume centers with dedicated multidisciplinary expertise in carcinoid management [3,158,161,162]. In appropriately selected patients, such comprehensive management improves symptoms, enables continuation of oncologic therapy, and extends survival.

## 11. Ethical and Quality-of-Life Considerations

Cardiac surgery in patients with cancer is among the most complex challenges in modern medicine, requiring decisions that extend beyond technical feasibility to include ethical judgment and QoL implications. Because these patients are often excluded from large, randomized trials, evidence is limited, and choices must rely on shared decision-making within an MDT, explicitly integrating patient goals and the oncologic context [3]. For patients with advanced or metastatic cancer, potential operative benefit must be balanced against perioperative burden, recovery time, complication risk, and the possibility of interrupting cancer therapy. Early integration of palliative care improves symptoms, QoL, and even survival, and can support consideration of medical or percutaneous alternatives when these are more aligned with patient priorities. In contrast, for early-stage malignancies with meaningful long-term prognosis, an invasive cardiac strategy may be justified when it facilitates timely and definitive oncologic treatment, in line with cardio-oncology principles aimed at minimizing unnecessary delays of cancer care.

Quality of life considerations remain central. Frailty and malnutrition/cachexia strongly predict poorer postoperative outcomes and smaller QoL gains after cardiac surgery, and should therefore be addressed during pre-operative assessment, counseling, and optimization [163,164,165]. Accordingly, shared decision-making with the patient and family should cover procedural risk, expected cardiac and oncologic outcomes, effects on the timing of chemotherapy or RT, and the patient’s personal values and preferences, ideally with palliative care input to support goals-of-care discussions [166]. Ethical principles—including autonomy, beneficence, non-maleficence, justice and proportionality—provide a structured framework for navigating these decisions. Ultimately, choices should honor the patient’s wishes and dignity and reflect what constitutes meaningful QoL for them.

## 12. Future Directions and Research Needs

Despite growing recognition of the overlap between CVD and malignancy, evidence guiding cardiac surgical management in cancer patients remains limited. Most available data are derived from retrospective cohort studies or national registries, with heterogeneity in cancer types, treatment stages, and surgical techniques. The exclusion of cancer patients from major cardiological randomized controlled trials has resulted in a lack of evidence-based recommendations for this specific population. The outcomes of cardiac surgery in patients with active cancer have not been extensively studied, primarily due to their historically limited access to cardiac surgical procedures. Randomized controlled trials are virtually absent, leaving clinicians to extrapolate from general cardiac surgery cohorts.

There is an urgent need to develop cancer-specific cardiac risk models that integrate oncologic parameters such as tumor stage, prognosis, anticipated treatment trajectory, and treatment-induced frailty alongside conventional surgical predictors. EuroSCORE II and the STS score remain widely used but consistently underestimate operative risk in patients with active cancer. Prospective validation of augmented scoring systems incorporating cancer variables would improve risk discrimination and patient counseling.

The role of transcatheter therapies also warrants further investigation. Although registry data suggest that outcomes after TAVR or PCI are comparable between cancer and non-cancer patients in the short term [167], long-term data are scarce, and the durability of valves or stents in patients undergoing chemotherapy or RT remains unclear. Similarly, the potential benefits of minimally invasive and robotic surgical approaches in reducing perioperative morbidity in oncology patients should be studied systematically.

Multidisciplinary cardio-oncology programs should be expanded and embedded in surgical pathways. Such programs facilitate MDT discussions, align cardiac and cancer treatment sequencing, and reduce futile interventions [168]. International collaboration is required to establish prospective registries dedicated to surgical outcomes in cancer patients, enabling benchmarking, harmonization of practice, and hypothesis generation for future clinical trials.

Finally, QoL-centered outcomes must be prioritized alongside survival. For many patients with advanced malignancies, the ability to maintain independence, avoid recurrent hospitalizations, and proceed with oncologic therapy is more meaningful than absolute survival gain. Future studies should incorporate validated QoL tools to better guide treatment strategies and shared decision-making.

## 13. Conclusions

Cardiac surgery in patients with cancer presents some of the most challenging dilemmas in modern cardiothoracic practice. These patients face a unique blend of risks related to hypercoagulability, bleeding, immunosuppression, frailty, and prior oncologic therapies. Conventional surgical risk models inadequately capture these complexities, and perioperative outcomes remain heterogeneous, heavily influenced by cancer stage and prognosis.

Nevertheless, carefully selected patients—particularly those with early-stage malignancy and curative treatment potential—cardiac surgery can be lifesaving, restore candidacy for oncologic therapy, and yield outcomes approaching those of non-cancer populations. Conversely, in advanced or metastatic disease, the risks of invasive intervention may outweigh benefits, emphasizing the importance of proportionality, patient values, and integration of palliative care principles.

Future progress hinges on the development of cancer-specific risk prediction models, systematic evaluation of transcatheter and minimally invasive strategies, and the establishment of robust cardio-oncology surgical registries. Above all, individualized, multidisciplinary care—anchored in shared decision-making—remains the cornerstone for optimizing outcomes. Cardiac surgeons, oncologists, and cardiologists must work together to navigate this intersection of specialties. By aligning surgical decision-making with oncologic trajectories and patient goals, clinicians can ensure that interventions are not only technically feasible but also ethically sound and clinically meaningful.

This schematic summarizes the key cancer-specific factors that complicate cardiothoracic surgical decision-making. These include increased thrombotic and bleeding risk related to cancer-associated hypercoagulability, anticoagulant exposure, and cardiopulmonary bypass; frailty and cachexia with impaired physiological reserve and delayed recovery; immunosuppression and heightened susceptibility to postoperative infection; and long-term sequelae of prior cancer therapies such as thoracic radiotherapy and cardiotoxic treatments. Additional considerations include cancer type and stage, timing and interaction with ongoing or future oncological therapies, anticipated quality of life impact, and patient preferences. Integration of these interrelated domains within a multidisciplinary cardio-oncology framework is essential to balance surgical benefit against perioperative risk and oncologic prognosis.

## Data Availability

No new data were created or analyzed in this study.

## Abbreviations

The following abbreviations are used in this manuscript:

ACS	Acute coronary syndrome
AS	Aortic stenosis
AF	Atrial fibrillation
CAT	Cancer-associated thrombosis
CAD	Coronary artery disease
CaHD	Cardiac carcinoid disease
CBP	Cardiopulmonary bypass
CLL	Chronic lymphocytic leukemia
CTRCD	Cancer-therapy-associated cardiac dysfunction
CV	Cardiovascular
CVD	Cardiovascular disease
CABG	Coronary artery bypass grafting
DAPT	Double antiplatelet therapy
DVT	Deep vein thrombosis
ESC	European Society of Cardiology
EACTS	European Association for Cardio-Thoracic Surgery
ERAS	Enhanced recovery after surgery
HM	Hematologic malignancies
ICI	Immune checkpoint inhibitor
LMWH	Low molecular weight heparin
MR	Mitral regurgitation
MDT	Multidisciplinary team
MIAVR	Minimally invasive aortic valve replacement
NK	Natural killer
NBTE	Non-bacterial thrombotic endocarditis
NSTEMI	Non-ST-elevation myocardial infarction
NETs	Neutrophil extracellular traps
OPCAB	Off-Pump Coronary Artery Bypass
PLT	Platelets
PE	Pulmonary embolism
PCI	Percutaneous Coronary Intervention
RT	Radiotherapy
RR	Relative risk
RVOT	Right Ventricular Outflow Tract
STS	Society of Thoracic Surgeons
STEMI	ST-elevation myocardial infarction
SAVR	Surgical aortic valve replacement
SVC	Superior vena cava
TAVR	Transcatheter aortic valve replacement
VATS	Video-assisted thoracoscopic surgery
VHD	Vascular heart disease
VTE	Venous Thromboembolism
QoL	Quality of life
WBC	White blood cells
IE	Infective endocarditis
IPC	Intermittent Pneumatic Compression

## References

[1] Phillips J.L., Currow D.C. (2010). Cancer as a chronic disease. Collegian.

[2] Shiels M.S., Freedman N.D., Haque A.T., de González A.B., Lipkowitz S., Lowy D.R., Pfeiffer R.M. (2025). US cancer deaths prevented due to survival improvements stratified by extent of disease, 2010–2019. JNCI J. Natl. Cancer Inst..

[3] Lyon A.R., López-Fernández T., Couch L.S., Asteggiano R., Aznar M.C., Bergler-Klein J., Boriani G., Cardinale D., Cordoba R., Cosyns B. (2022). 2022 ESC Guidelines on cardio-oncology developed in collaboration with the European Hematology Association (EHA), the European Society for Therapeutic Radiology and Oncology (ESTRO) and the International Cardio-Oncology Society (IC-OS). Eur. Heart J. Cardiovasc. Imaging.

[4] Koene R.J., Prizment A.E., Blaes A., Konety S.H. (2016). Shared Risk Factors in Cardiovascular Disease and Cancer. Circulation.

[5] Lorusso R., Vizzardi E., Johnson D.M., Mariscalco G., Sciatti E., Maessen J., Bidar E., Gelsomino S. (2018). Cardiac surgery in adult patients with remitted or active malignancies: A review of preoperative screening, surgical management and short- and long-term postoperative results. Eur. J. Cardio-Thorac. Surg..

[6] Chandiramani A., Ali J.M. (2025). Frailty in Cardiac Surgery—Assessment Tools, Impact on Outcomes, and Optimisation Strategies: A Narrative Review. J. Cardiovasc. Dev. Dis..

[7] https://cancercontrol.cancer.gov/ocs/definitions.

[8] Jaworski C., Mariani J.A., Wheeler G., Kaye D.M. (2013). Cardiac Complications of Thoracic Irradiation. J. Am. Coll. Cardiol..

[9] Heidenreich P.A., Kapoor J.R. (2009). Radiation induced heart disease: Systemic disorders in heart disease. Heart.

[10] Mennander A.A., Nielsen S.J., Huhtala H., Dellgren G., Hansson E.C., Jeppsson A. (2022). History of cancer and survival after coronary artery bypass grafting: Experiences from the SWEDEHEART registry. J. Thorac. Cardiovasc. Surg..

[11] Guha A., Dey A.K., Kalra A., Gumina R., Lustberg M., Lavie C.J., Sabik J.F., Addison D. (2020). Coronary Artery Bypass Grafting in Cancer Patients: Prevalence and Outcomes in the United States. Mayo Clin. Proc..

[12] Handa N., McGregor C.G., Danielson G.K., Daly R.C., Dearani J.A., Mullany C.J., Orszulak T.A., Schaff H.V., Zehr K.J., Anderson B.J. (2001). Valvular heart operation in patients with previous mediastinal radiation therapy. Ann. Thorac. Surg..

[13] Gujral D.M., Lloyd G., Bhattacharyya S. (2016). Radiation-induced valvular heart disease. Heart.

[14] Fernández-Cruz A., Muñoz P., Sandoval C., Fariñas C., Gutiérrez-Cuadra M., Pericás Pulido J.M., Miró J.M., Goenaga-Sánchez M.Á., de Alarcón A., Bonache-Bernal F. (2017). Infective endocarditis in patients with cancer: A consequence of invasive procedures or a harbinger of neoplasm? A prospective, multicenter cohort. Medicine.

[15] Grable C., Yusuf S.W., Song J., Viola G.M., Ulhaq O., Banchs J., Jensen C.T., Goel H., Hassan S.A. (2021). Characteristics of infective endocarditis in a cancer population. Open Heart.

[16] Huba M., Hussain F., Guntaka S., Paracha A., Sathe P., Parikh B., Noyelle M., Durrani U., Patel H., John V. (2025). Non-bacterial thrombotic endocarditis in ovarian cancer: A systematic review. Gynecol. Oncol. Rep..

[17] Parker N., Atallah R., Ojile N., Chamoun K., Nehme F., Vindhyal M. (2020). Nonbacterial Thrombotic Endocarditis. Kans. J. Med..

[18] Quintero-Martinez J.A., Hindy J.R., El Zein S., Michelena H.I., Nkomo V.T., DeSimone D.C., Baddour L.M. (2022). Contemporary demographics, diagnostics and outcomes in non-bacterial thrombotic endocarditis. Heart.

[19] Ahmed O., E King N., Qureshi M.A., Choudhry A.A., Osama M., Zehner C., Ali A., Hamzeh I.R., Palaskas N.L., Thompson K.A. (2024). Non-bacterial thrombotic endocarditis: A clinical and pathophysiological reappraisal. Eur. Hear. J..

[20] Keramida K., Kariki O., Angelopoulou E., Kalafatis I., Lafaras C., Letsas K.P., Michalopoulou H., Saplaouras A., Tampakis K., Tsekoura D. (2025). Arrhythmias, conduction disorders and sudden cardiac death in cancer patients and survivors: Expert opinion of the working groups on cardio-oncology and on electrophysiology of the hellenic cardiac society. Cardio-Oncology.

[21] Farmakis D., Parissis J., Filippatos G. (2014). Insights into onco-cardiology: Atrial fibrillation in cancer. J. Am. Coll. Cardiol..

[22] von Ballmoos M.C.W., Hui D.S., Mehaffey J.H., Malaisrie S.C., Vardas P.N., Gillinov A.M., Sundt T.M., Badhwar V. (2024). The Society of Thoracic Surgeons 2023 Clinical Practice Guidelines for the Surgical Treatment of Atrial Fibrillation. Ann. Thorac. Surg..

[23] Adams M.J., Hardenbergh P.H., Constine L.S., Lipshultz S.E. (2003). Radiation-associated cardiovascular disease. Crit. Rev. Oncol. Hematol..

[24] Burazor I., Imazio M., Markel G., Adler Y. (2013). Malignant pericardial effusion. Cardiology.

[25] Maisch B., Ristic A., Pankuweit S. (2010). Evaluation and Management of Pericardial Effusion in Patients with Neoplastic Disease. Prog. Cardiovasc. Dis..

[26] Ning M.S., Tang L., Gomez D.R., Xu T., Luo Y., Huo J., Mouhayar E., Liao Z. (2017). Incidence and Predictors of Pericardial Effusion After Chemoradiation Therapy for Locally Advanced Non-Small Cell Lung Cancer. Int. J. Radiat. Oncol..

[27] Lee J., Kim K., Gwak S.-Y., Lee H.-J., Cho I., Hong G.-R., Ha J.-W., Shim C.Y. (2024). Pericardiocentesis versus window formation in malignant pericardial effusion: Trends and outcomes. Heart.

[28] Bertog S.C., Thambidorai S.K., Parakh K., Schoenhagen P., Ozduran V., Houghtaling P.L., Lytle B.W., Blackstone E.H., Lauer M.S., Klein A.L. (2004). Constrictive pericarditis: Etiology and cause-specific survival after pericardiectomy. J. Am. Coll. Cardiol..

[29] Ling L.H., Oh J.K., Schaff H.V., Danielson G.K., Mahoney D.W., Seward J.B., Tajik A.J. (1999). Constrictive pericarditis in the modern era: Evolving clinical spectrum and impact on outcome after pericardiectomy. Circulation.

[30] Tie H., Zhu J., Akin S., Allen L.A., Huang B., Martens S., Welp H., Simpkin A., Shi R., Wu Q. (2023). Characteristics and Outcome of Patients with a History of Cancer Undergoing Durable Left Ventricular Assist Device Implantation. Circ. Heart Fail..

[31] Mulzer J., Müller M., Schoenrath F., Falk V., Potapov E., Knierim J. (2022). Left Ventricular Assist Device Implantation in Cancer-Therapy-Related Heart Failure. Life.

[32] Schlam I., Lee A.Y., Li S., Sheikh F.H., Zaghlol R., Basyal B., Gallagher C., Molina E., Mahr C., Cheng R.K. (2021). Left Ventricular Assist Devices in Patients with Active Malignancies. JACC CardioOncology.

[33] Khorana A.A., Mackman N., Falanga A., Pabinger I., Noble S., Ageno W., Moik F., Lee A.Y.Y. (2022). Cancer-associated venous thromboembolism. Nat. Rev. Dis. Primers.

[34] Heit J.A., Silverstein M.D., Mohr D.N., Petterson T.M., O’Fallon W.M., Melton L.J. (2000). Risk factors for deep vein thrombosis and pulmonary embolism: A population-based case-control study. Arch. Intern. Med..

[35] Falanga A., Marchetti M., Russo L. (2015). The mechanisms of cancer-associated thrombosis. Thromb. Res..

[36] Fuchs T.A., Brill A., Wagner D.D. (2012). Neutrophil Extracellular Trap (NET) Impact on Deep Vein Thrombosis. Arter. Thromb. Vasc. Biol..

[37] Ho K.M., Bham E., Pavey W. (2015). Incidence of Venous Thromboembolism and Benefits and Risks of Thromboprophylaxis After Cardiac Surgery: A Systematic Review and Meta-Analysis. Am. Heart Assoc. J..

[38] Khoury H., Lyons R., Sanaiha Y., Rudasill S., Shemin R.J., Benharash P. (2020). Deep Venous Thrombosis and Pulmonary Embolism in Cardiac Surgical Patients. Ann. Thorac. Surg..

[39] Khorana A.A., Francis C.W. (2018). Risk prediction of cancer-associated thrombosis: Appraising the first decade and developing the future. Thromb. Res..

[40] Shahbaz A., Wannakuwatte R.A., Mohammed C., Alzarooni A., Pendem H., Majeed F., Kuruba V., Metry S., Mahajan T., Reza H. (2024). Transformative Deep Vein Thrombosis Prophylaxis with Sequential Compression Devices in the Care of Hospitalized Patients. Cureus.

[41] Ahmed A., Koster A., Lance M., Milojevic M. (2024). European guidelines on perioperative venous thromboembolism prophylaxis: Cardiovascular surgery. Eur. J. Cardio-Thoracic Surg..

[42] Sandner S., Gaudino M., Agewall S., Bonalumi G., Bonaros N., Czerny M., Jeppsson A., Milojevic M., Niessner A., Parolari A. (2025). Antithrombotic therapy after coronary artery bypass graft surgery: A Clinical Consensus Statement of the ESC Working Group on Cardiovascular Surgery, the ESC Working Group on Cardiovascular Pharmacotherapy, and the European Association for Cardio-Thoracic Surgery (EACTS). Eur. Heart J..

[43] Despotis G.J., Avidan M.S., Hogue C.W. (2001). Mechanisms and attenuation of hemostatic activation during extracorporeal circulation. Ann. Thorac. Surg..

[44] Biancari F., Mikkola R., Heikkinen J., Lahtinen J., Airaksinen K.J., Juvonen T. (2011). Estimating the risk of complications related to re-exploration for bleeding after adult cardiac surgery: A systematic review and meta-analysis. Eur. J. Cardio-Thoracic Surg..

[45] Glassman A.B. (1998). Hemostatic abnormalities associated with cancer and its therapy. Ann. Clin. Lab. Sci..

[46] Wells M., Raja M., Rahman S. (2022). Point-of-care viscoelastic testing. BJA Educ..

[47] Myles P.S., Smith J.A., Forbes A., Silbert B., Jayarajah M., Painter T., Cooper D.J., Marasco S., McNeil J., Bussières J.S. (2017). Tranexamic Acid in Patients Undergoing Coronary-Artery Surgery. N. Engl. J. Med..

[48] Clegg A., Young J., Iliffe S., Rikkert M.O., Rockwood K. (2013). Frailty in elderly people. Lancet.

[49] Handforth C., Clegg A., Young C., Simpkins S., Seymour M.T., Selby P.J., Young J. (2014). The prevalence and outcomes of frailty in older cancer patients: A systematic review. Ann. Oncol..

[50] Weinländer P., Hadzibegovic S., Porthun J., Kretzler L., Evertz R., Lena A., Lück L., Hella J.L., Wilkenshoff U., Stroux A. (2025). The Prognostic Role of Frailty and Its Recognition With Simple FRAIL and Fried Frailty Questionnaires in Advanced Cancer Patients. J. Cachexia Sarcopenia Muscle.

[51] Lee J.A., Yanagawa B., An K.R., Arora R.C., Verma S., Friedrich J.O., on behalf of the Canadian Cardiovascular Surgery Meta-Analysis Working Group (2021). Frailty and pre-frailty in cardiac surgery: A systematic review and meta-analysis of 66,448 patients. J. Cardiothorac. Surg..

[52] Fried L.P., Tangen C.M., Walston J., Newman A.B., Hirsch C., Gottdiener J., Seeman T., Tracy R., Kop W.J., Burke G. (2001). Frailty in older adults: Evidence for a phenotype. J. Gerontol. Ser. A Biol. Sci. Med. Sci..

[53] Rockwood K., Song X., MacKnight C., Bergman H., Hogan D.B., McDowell I., Mitnitski A. (2005). A global clinical measure of fitness and frailty in elderly people. CMAJ Can. Med. Assoc. J. J. De L’association Medicale Can..

[54] Mitnitski A.B., Mogilner A.J., Rockwood K. (2001). Accumulation of deficits as a proxy measure of aging. Sci. World J..

[55] von Haehling S., Anker S.D. (2010). Cachexia as a major underestimated and unmet medical need: Facts and numbers. J. Cachex-Sarcopenia Muscle.

[56] Guigoz Y. (2006). The Mini Nutritional Assessment (MNA) review of the literature—What does it tell us?. J. Nutr. Health Aging.

[57] Prado C.M., Lieffers J.R., McCargar L.J., Reiman T., Sawyer M.B., Martin L., Baracos V.E. (2008). Prevalence and clinical implications of sarcopenic obesity in patients with solid tumours of the respiratory and gastrointestinal tracts: A population-based study. Lancet Oncol..

[58] Mastroiacovo G., Bonomi A., Ludergnani M., Franchi M., Maragna R., Pirola S., Baggiano A., Caglio A., Pontone G., Polvani G. (2022). Is EuroSCORE II still a reliable predictor for cardiac surgery mortality in 2022? A retrospective study study. Eur. J. Cardio-Thoracic Surg..

[59] Bowdish M.E., D’agostino R.S., Thourani V.H., Schwann T.A., Krohn C., Desai N., Shahian D.M., Fernandez F.G., Badhwar V. (2021). STS Adult Cardiac Surgery Database: 2021 Update on Outcomes, Quality, and Research. Ann. Thorac. Surg..

[60] Bezu L., Öksüz D.A., Bell M., Buggy D., Diaz-Cambronero O., Enlund M., Forget P., Gupta A., Hollmann M.W., Ionescu D. (2024). Perioperative Immunosuppressive Factors during Cancer Surgery: An Updated Review. Cancers.

[61] Rodríguez-López J.M., Iglesias-González J.L., Lozano-Sánchez F.S., Palomero-Rodríguez M.Á., Sánchez-Conde P. (2022). Inflammatory Response, Immunosuppression and Arginase Activity after Cardiac Surgery Using Cardiopulmonary Bypass. J. Clin. Med..

[62] Mao G., Chen W., Wang L., Zhao S., Zang F. (2025). Clinical risk factors for postoperative infection in adult cardiac surgery with cardiopulmonary bypass: A retrospective study. Infect. Prev. Pract..

[63] Barbato R., Ferraresi B., Chello M., Strumia A., Gagliardi I., Loreni F., Mattei A., Santarpino G., Carassiti M., Grigioni F. (2025). Length and Type of Antibiotic Prophylaxis for Infection Prevention in Adults Patient in the Cardiac Surgery Intensive Care Unit: A Narrative Review. Antibiotics.

[64] Desai M.Y., Windecker S., Lancellotti P., Bax J.J., Griffin B.P., Cahlon O., Johnston D.R. (2019). Prevention, Diagnosis, and Management of Radiation-Associated Cardiac Disease. JACC.

[65] Handa N., McGregor C.G., Danielson G.K., Orszulak T.A., Mullany C.J., Daly R.C., Dearani J.A., Anderson B.J., Puga F.J. (1999). Coronary artery bypass grafting in patients with previous mediastinal radiation therapy. J. Thorac. Cardiovasc. Surg..

[66] Dolmaci O.B., Farag E.S., Boekholdt S.M., Boven W.J.P., Kaya A. (2019). Outcomes of cardiac surgery after mediastinal radiation therapy: A single-center experience. J. Card. Surg..

[67] Nashef S.A., Roques F., Sharples L.D., Nilsson J., Smith C., Goldstone A.R., Lockowandt U. (2012). EuroSCORE II. Eur. J. Cardio-Thorac. Surg. Off. J. Eur. Assoc. Cardio-Thorac. Surg..

[68] Shahian D.M., O’Brien S.M., Filardo G., Ferraris V.A., Haan C.K., Rich J.B., Normand S.L., DeLong E.R., Shewan C.M., Dokholyan R.S. (2009). The Society of Thoracic Surgeons 2008 cardiac surgery risk models: Part 1—coronary artery bypass grafting surgery. Ann. Thorac. Surg..

[69] Afilalo J., Mottillo S., Eisenberg M.J., Alexander K.P., Noiseux N., Perrault L.P., Morin J.-F., Langlois Y., Ohayon S.M., Monette J. (2012). Addition of Frailty and Disability to Cardiac Surgery Risk Scores Identifies Elderly Patients at High Risk of Mortality or Major Morbidity. Circ. Cardiovasc. Qual. Outcomes.

[70] Kim C.H., Kang Y., Kim J.S., Sohn S.H., Hwang H.Y. (2022). Association Between the Frailty Index and Clinical Outcomes after Coronary Artery Bypass Grafting. J. Chest Surg..

[71] Shaw J.F., Budiansky D., Sharif F., McIsaac D.I. (2022). The Association of Frailty with Outcomes after Cancer Surgery: A Systematic Review and Metaanalysis. Ann. Surg. Oncol..

[72] Pushparaji B., Donisan T., Balanescu D.V., Park J.K., Monlezun D.J., Ali A., Inanc I.H., Caballero J., Cilingiroglu M., Marmagkiolis K. (2023). Coronary Revascularization in Patients with Cancer. Current treatment options in cardiovascular medicine. Curr. Treat. Options Cardiovasc. Med..

[73] Hanna T.P., King W.D., Thibodeau S., Jalink M., Paulin G.A., Harvey-Jones E., O’Sullivan D.E., Booth C.M., Sullivan R., Aggarwal A. (2020). Mortality due to cancer treatment delay: Systematic review and meta-analysis. BMJ.

[74] Mallick S., Ebrahimian S., Sakowitz S., Le N., Bakhtiyar S.S., Benharash P. (2025). Evaluation of the Timing to Noncardiac Surgery following Cardiac Operations: A National Analysis. JACC Adv..

[75] Yeginsu A., Vayvada M., Karademir B.C., Erkılınç A., Tasci A.E., Buyukbayrak F., Gurcu E., Kutlu C.A. (2018). Combined Off-Pump Coronary Artery Bypass Grafting and Lung Resection in Patients with Lung Cancer Accompanied by Coronary Artery Disease. Braz. J. Cardiovasc. Surg..

[76] Andrushchuk U., Ostrovsky Y., Krasny S., Polyakov S., Zharkov V., Rolevich A., Kurganovich S., Krutau V., Amelchanka S.G. (2017). Simultaneous or staged surgery in patients with kidney tumors and concomitant cardiac disease. Central Eur. J. Urol..

[77] Ateş M.Ş., Demirözü Z.T., Erus S., Aksoy E., Özer K.B., Gürkahraman S., Cesur E.E., Tanju S. (2025). The outcomes of concomitant off-pump coronary artery bypass grafting and pulmonary operations. Turk. J. Thorac. Cardiovasc. Surg..

[78] Chen F., Gao S., Xia W., Wang M., Zhang W., Xu Y. (2025). Perioperative safety of simultaneous pulmonary resection and off-pump coronary artery bypass grafting: A retrospective analysis from a single institution. J. Thorac. Dis..

[79] Tourmousoglou C.E., Apostolakis E., Dougenis D. (2014). Simultaneous occurrence of coronary artery disease and lung cancer: What is the best surgical treatment strategy?. Interdiscip. Cardiovasc. Thorac. Surg..

[80] American Society of Breast Surgeons Resource Guide: Preoperative Management of Patients Treated with Neoadjuvant Systemic Therapy. https://www.breastsurgeons.org/docs/statements/asbrs-nst.pdf.

[81] Cullinane C., Shrestha A., Al Maksoud A., Rothwell J., Evoy D., Geraghty J., McCartan D., McDermott E.W., Prichard R.S. (2021). Optimal timing of surgery following breast cancer neoadjuvant chemotherapy: A systematic review and meta-analysis. Eur. J. Surg. Oncol..

[82] Perioperative Use of Avastin. https://medically.roche.com/global/en/medinfo/avastin/perioperative-use-of-avastin.html?utm_source=chatgpt.com.

[83] Highlights of Prescribing Information. https://www.gene.com/download/pdf/avastin_prescribing.pdf.

[84] Brown J.A., Aranda-Michel E., Kilic A., Serna-Gallegos D., Bianco V., Thoma F.W., Sultan I. (2022). Impact of Thoracic Radiation on Patients Undergoing Cardiac Surgery. Semin. Thorac. Cardiovasc. Surg..

[85] Ejiofor J.I., Val F.R.-D., Nohria A., Norman A., McGurk S., Aranki S.F., Shekar P., Cohn L.H., Kaneko T. (2017). The risk of reoperative cardiac surgery in radiation-induced valvular disease. J. Thorac. Cardiovasc. Surg..

[86] Jiritano F., Matteucci M., Guareschi A., Fina D., Vizzardi E., Mariscalco G., Sciatti E., Lorusso R. (2019). Cardiac surgery in patients with malignancy: A literature review and recommendations for perioperative management. G. Ital. Di Cardiol..

[87] Bonelli A., Lorusso R., Paris S., Troise G., Mohammed A.H., Bursi F., Faggiano P. (2021). Active cancer and cardiac surgery: Possible scenarios in patient decision-making. Monaldi Arch. Chest Dis..

[88] Płońska-Gościniak E., Piotrowski G., Wojakowski W., Gościniak P., Olszowska M., Lesiak M., Klotzka A., Grygier M., Deja M., Kasprzak J.D. (2023). Management of valvular heart disease in patients with cancer: Multidisciplinary team, cancer-therapy related cardiotoxicity, diagnosis, transcatheter intervention, and cardiac surgery. Expert opinion of the Association on Valvular Heart Disease, Association of Cardiovascular Interventions, and Working Group on Cardiac Surgery of the Polish Cardiac Society. Kardiol. Pol..

[89] Curigliano G., Lenihan D., Fradley M., Ganatra S., Barac A., Blaes A., Herrmann J., Porter C., Lyon A.R., Lancellotti P. (2020). Management of cardiac disease in cancer patients throughout oncological treatment: ESMO consensus recommendations. Ann. Oncol..

[90] Batchelor W.B., Anwaruddin S., Wang D.D., Perpetua E.M., Krishnaswami A., Velagapudi P., Wyman J.F., Fullerton D., Keegan P., Phillips A. (2023). The Multidisciplinary Heart Team in Cardiovascular Medicine: Current Role and Future Challenges. JACC Adv..

[91] Grant M.C., Crisafi C., Alvarez A., Arora R.C., Brindle M.E., Chatterjee S., Ender J., Fletcher N., Gregory A.J., Gunaydin S. (2024). Perioperative Care in Cardiac Surgery: A Joint Consensus Statement by the Enhanced Recovery After Surgery (ERAS) Cardiac Society, ERAS International Society, and The Society of Thoracic Surgeons (STS). Ann. Thorac. Surg..

[92] Leong D.P., Cirne F., Aghel N., Baro Vila R.C., Cavalli G.D., Ellis P.M., Healey J.S., Whitlock R., Khalaf D., Mian H. (2023). Cardiac Interventions in Patients with Active, Advanced Solid and Hematologic Malignancies: *JACC: CardioOncology* State-of-the-Art Review. JACC CardioOncol..

[93] Chakrabarti S., Peterson C.Y., Sriram D., Mahipal A. (2020). Early stage colon cancer: Current treatment standards, evolving paradigms, and future directions. World J. Gastrointest. Oncol..

[94] Ducreux M., Cuhna A.S., Caramella C., Hollebecque A., Burtin P., Goéré D., Seufferlein T., Haustermans K., Van Laethem J.L., Conroy T. (2015). Cancer of the pancreas: ESMO Clinical Practice Guidelines for diagnosis, treatment and follow-up. Ann. Oncol..

[95] Staab J., Cotter E., Kidd B., Wallisch W.J., Flynn B.C. (2020). Review and Update: Hematologic Malignancies and Adult Cardiac Surgery. J. Cardiothorac. Vasc. Anesth..

[96] Plumereau F., Pinaud F., Roch A., Baufreton C. (2014). Do patients with haematological malignancy who need cardiopulmonary bypass have a short-term higher mortality or a higher chance of disease progression?. Interdiscip. Cardiovasc. Thorac. Surg..

[97] Nguyen A., Schaff H.V., Arghami A., Bagameri G., Cicek M.S., Crestanello J.A., Daly R.C., Greason K.L., Pochettino A., Rowse P.G. (2021). Impact of Hematologic Malignancies on Outcome of Cardiac Surgery. Ann. Thorac. Surg..

[98] Madrigal J., Tran Z., Hadaya J., Sanaiha Y., Benharash P. (2022). Impact of Chronic Lymphocytic Leukemia on Outcomes and Readmissions After Cardiac Operations. Ann. Thorac. Surg..

[99] Zhu Y., Toth A.J., Lowry A.M., Blackstone E.H., Hill B.T., Mick S.L. (2018). Cardiac Surgery Outcomes in Patients with Chronic Lymphocytic Leukemia. Ann. Thorac. Surg..

[100] Yi M., Hu L., Zhou J., Ge Y., Su C., Yang F. (2025). Impact of hospital variation in hematologic malignancy patient proportions on outcomes of chronic lymphocytic leukemia patients undergoing cardiac surgery: Insights from nationwide data analysis. Cardio-Oncology.

[101] Cunningham R., Yoo S.G.K., Brescia A.A., Oetjen K.A., Pusic I., Mitchell J.D. (2025). Severe Aortic Stenosis and Chronic Myeloid Leukemia: Considerations for Valve Management. JACC CardioOncology.

[102] Palmieri V., Vietri M.T., Montalto A., Montisci A., Donatelli F., Coscioni E., Napoli C. (2023). Cardiotoxicity, Cardioprotection, and Prognosis in Survivors of Anticancer Treatment Undergoing Cardiac Surgery: Unmet Needs. Cancers.

[103] Iliescu C.A., Grines C.L., Herrmann J., Yang E.H., Cilingiroglu M., Charitakis K., Hakeem A., Toutouzas K.P., Leesar M.A., Marmagkiolis K. (2016). SCAI Expert consensus statement: Evaluation, management, and special considerations of cardio-oncology patients in the cardiac catheterization laboratory (endorsed by the cardiological society of india, and sociedad Latino Americana de Cardiologıa intervencionista). Catheter. Cardiovasc. Interv. Off. J. Soc. Card. Angiogr. Interv..

[104] Falanga A., Leader A., Ambaglio C., Bagoly Z., Castaman G., Elalamy I., Lecumberri R., Niessner A., Pabinger I., Szmit S. (2022). EHA Guidelines on Management of Antithrombotic Treatments in Thrombocytopenic Patients With Cancer. HemaSphere.

[105] Doolub G., Mamas M.A. (2022). Percutaneous Coronary Angioplasty in Patients with Cancer: Clinical Challenges and Management Strategies. J. Pers. Med..

[106] Campos C.M., Mehran R., Capodanno D., Owen R., Windecker S., Varenne O., Stone G.W., Valgimigli M., Hajjar L.A., Filho R.K. (2024). Risk Burden of Cancer in Patients Treated With Abbreviated Dual Antiplatelet Therapy After PCI: Analysis of Multicenter Controlled High-Bleeding Risk Trials. Circ. Cardiovasc. Interv..

[107] Gent D.G., Rebecca D. (2023). The 2022 European Society of Cardiology Cardio-oncology Guidelines in Focus. Eur. Cardiol..

[108] Virani S.S., Newby L.K., Arnold S.V., Bittner V., Brewer L.C., Demeter S.H., Dixon D.L., Fearon W.F., Hess B., Johnson H.M. (2023). 2023 AHA/ACC/ACCP/ASPC/NLA/PCNA Guideline for the Management of Patients with Chronic Coronary Disease: A Report of the American Heart Association/American College of Cardiology Joint Committee on Clinical Practice Guidelines. Circulation.

[109] DeZorzi C. (2018). Radiation-Induced Coronary Artery Disease and Its Treatment: A Quick Review of Current Evidence. Cardiol. Res. Pract..

[110] Li R., Prastein D.J. (2025). Short-Term Outcomes of Patients with Non-Metastatic Malignant Solid Tumor after Coronary Artery Bypass Grafting: A Population Based Study of National/Nationwide Inpatient Sample From 2015 to 2020. Braz. J. Cardiovasc. Surg..

[111] Belzile-Dugas E., Fremes S.E., Eisenberg M.J. (2023). Radiation-Induced Aortic Stenosis. JACC.

[112] Otto C.M., Nishimura R.A., Bonow R.O., Carabello B.A., Erwin J.P., Gentile F., Jneid H., Krieger E.V., Mack M., McLeod C. (2021). 2020 ACC/AHA Guideline for the Management of Patients with Valvular Heart Disease: A Report of the American College of Cardiology/American Heart Association Joint Committee on Clinical Practice Guidelines. Circulation.

[113] Praz F., Borger M.A., Lanz J., Marin-Cuartas M., Abreu A., Adamo M., Marsan N.A., Barili F., Bonaros N., Cosyns B. (2025). 2025 ESC/EACTS Guidelines for the management of valvular heart disease: Developed by the task force for the management of valvular heart disease of the European Society of Cardiology (ESC) and the European Association for Cardio-Thoracic Surgery (EACTS). Eur. Heart J..

[114] Elbadawi A., Albaeni A., Elgendy I.Y., Ogunbayo G.O., Jimenez E., Cornwell L., Chatterjee A., Khalife W., Alkhouli M., Kapadia S.R. (2020). Transcatheter Versus Surgical Aortic Valve Replacement in Patients with Prior Mediastinal Radiation. JACC Cardiovasc. Interv..

[115] Zhang D., Guo W., Al-Hijji M.A., El Sabbagh A., Lewis B.R., Greason K., Sandhu G.S., Eleid M.F., Holmes D.R., Herrmann J. (2019). Outcomes of Patients with Severe Symptomatic Aortic Valve Stenosis After Chest Radiation: Transcatheter Versus Surgical Aortic Valve Replacement. J. Am. Hear. Assoc..

[116] Otto C.M., Kumbhani D.J., Alexander K.P., Calhoon J.H., Desai M.Y., Kaul S., Lee J.C., Ruiz C.E., Vassileva C.M. (2017). 2017 ACC Expert Consensus Decision Pathway for Transcatheter Aortic Valve Replacement in the Management of Adults with Aortic Stenosis. JACC.

[117] Vahanian A., Beyersdorf F., Praz F., Milojevic M., Baldus S., Bauersachs J., Capodanno D., Conradi L., De Bonis M., De Paulis R. (2022). 2021 ESC/EACTS Guidelines for the management of valvular heart disease. EuroIntervention J. Eur. Collab. Work. Group Interv. Cardiol. Eur. Soc. Cardiol..

[118] Nauffal V., Bay C., Shah P.B., Sobieszczyk P.S., Kaneko T., O’gAra P., Nohria A. (2021). Short-Term Outcomes of Transcatheter Versus Isolated Surgical Aortic Valve Replacement for Mediastinal Radiation-Associated Severe Aortic Stenosis. Circ. Cardiovasc. Interv..

[119] Ebrahimian S., Chervu N., Balian J., Mallick S., Yang E.H., Ziaeian B., Aksoy O., Benharash P. (2024). Timing of Noncardiac Surgery Following Transcatheter Aortic Valve Replacement: A National Analysis. JACC Cardiovasc. Interv..

[120] Okuno T., Demirel C., Tomii D., Erdoes G., Heg D., Lanz J., Praz F., Zbinden R., Reineke D., Räber L. (2022). Risk and Timing of Noncardiac Surgery After Transcatheter Aortic Valve Implantation. JAMA Netw. Open.

[121] Aikawa T., Kuno T., Malik A.H., Briasoulis A., Kolte D., Kampaktsis P.N., Latib A. (2023). Transcatheter Aortic Valve Replacement in Patients With or Without Active Cancer. J. Am. Hear. Assoc..

[122] Bendary A., Ramzy A., Bendary M., Salem M. (2020). Transcatheter aortic valve replacement in patients with severe aortic stenosis and active cancer: A systematic review and meta-analysis. Open Heart.

[123] Saberian P., Contreras R., Gurram A., Nasrollahizadeh A., Keetha N.R., Nguyen A.L., Nayak S.S., Keivanlou M., Hashemi M., Amini-Salehi E. (2025). Clinical Outcomes and Prognostic Implications of TAVR in Patients with Active Cancer: A Meta-Analysis. Clin. Cardiol..

[124] Ameen D., Thakker N., Contreras R., Hashemi S.M., Nasrollahizadeh A., Saberian P., Kuriyakose D., Amini-Salehi E., Keetha N.R., Nayak S.S. (2025). Clinical outcomes of transcatheter aortic valve replacement in patients with radiation-induced aortic stenosis: A systematic review and meta-analysis. Front. Cardiovasc. Med..

[125] Glauber M., Ferrarini M., Miceli A. (2015). Minimally invasive aortic valve surgery: State of the art and future directions. Ann. Cardiothorac. Surg..

[126] Almeida A.S., Ceron R.O., Anschau F., de Oliveira J.B., Neto T.C.L., Rode J., Rey R.A.W., Lira K.B., Delvaux R.S., de Souza R.O.R. (2022). Conventional Versus Minimally Invasive Aortic Valve Replacement Surgery: A Systematic Review, Meta-Analysis, and Meta-Regression. Innov. Technol. Tech. Cardiothorac. Vasc. Surg..

[127] Lorenzo-Esteller L., Ramos-Polo R., Riverola A.P., Morillas H., Berdejo J., Pernas S., Pomares H., Asiain L., Garay A., Pérez E.M. (2024). Pericardial Disease in Patients with Cancer: Clinical Insights on Diagnosis and Treatment. Cancers.

[128] Labbé C., Tremblay L., Lacasse Y. (2015). Pericardiocentesis versus Pericardiotomy for Malignant Pericardial Effusion: A Retrospective Comparison. Curr. Oncol..

[129] Allama A.M. (2016). Video-assisted thoracoscopic pleuro-pericardial window for recurrent massive pericardial effusion in patients with known malignancy. J. Egypt. Soc. Cardio-Thoracic Surg..

[130] Georghiou G.P., Stamler A., Sharoni E., Fichman-Horn S., Berman M., Vidne B.A., Saute M. (2005). Video-Assisted Thoracoscopic Pericardial Window for Diagnosis and Management of Pericardial Effusions. Ann. Thorac. Surg..

[131] Langdon S.E., Seery K., Kulik A. (2016). Contemporary outcomes after pericardial window surgery: Impact of operative technique. J. Cardiothorac. Surg..

[132] Kim S.M., Lee J.H., Chung S.R., Sung K., Kim W.S., Cho Y.H. (2024). Pericardial Window Operation in Oncology Patients: Analysis of Long-Term Survival and Prognostic Factors. J. Chest Surg..

[133] Celik S., Celik M., Aydemir B., Tanrıkulu H., Okay T., Tanrikulu N. (2012). Surgical properties and survival of a pericardial window via left minithoracotomy for benign and malignant pericardial tamponade in cancer patients. World J. Surg. Oncol..

[134] Adler Y., Charron P., Imazio M., Badano L., Barón-Esquivias G., Bogaert J., Brucato A., Gueret P., Klingel K., Lionis C. (2015). 2015 ESC Guidelines for the diagnosis and management of pericardial diseases: The Task Force for the Diagnosis and Management of Pericardial Diseases of the European Society of Cardiology (ESC)Endorsed by: The European Association for Cardio-Thoracic Surgery (EACTS). Eur. Heart J..

[135] Choi M.S., Jeong D.S., Oh J.K., Chang S.-A., Park S.-J., Chung S. (2019). Long-term results of radical pericardiectomy for constrictive pericarditis in Korean population. J. Cardiothorac. Surg..

[136] Pahwa S., Crestanello J., Miranda W., Bernabei A., Polycarpou A., Schaff H., Dearani J., Stulak J., Pochettino A., Daly R. (2021). Outcomes of pericardiectomy for constrictive pericarditis following mediastinal irradiation. J. Card. Surg..

[137] Szabó G., Schmack B., Bulut C., Soós P., Weymann A., Stadtfeld S., Karck M. (2013). Constrictive pericarditis: Risks, aetiologies and outcomes after total pericardiectomy: 24 years of experience. Eur. J. Cardio-Thoracic Surg..

[138] Wright K., Digby G.C., Gyawali B., Jad R., Menard A., Moraes F.Y., Wijeratne D.T. (2023). Malignant Superior Vena Cava Syndrome: A Scoping Review. J. Thorac. Oncol..

[139] Chow R., Simone C.B., Rimner A. (2024). Management of malignant superior vena cava syndrome. Ann. Palliat. Med..

[140] Aung E.Y.-S., Khan M., Williams N., Raja U., Hamady M. (2022). Endovascular Stenting in Superior Vena Cava Syndrome: A Systematic Review and Meta-analysis. Cardiovasc. Interv. Radiol..

[141] Uberoi R. (2006). Quality Assurance Guidelines for Superior Vena Cava Stenting in Malignant Disease. Cardiovasc. Interv. Radiol..

[142] Azizi A.H., Shafi I., Zhao M., Chatterjee S., Roth S.C., Singh M., Lakhter V., Bashir R. (2021). Endovascular therapy for superior vena cava syndrome: A systematic review and meta-analysis. eClinicalMedicine.

[143] Lanuti M., De Delva P.E., Gaissert H.A., Wright C.D., Wain J.C., Allan J.S., Donahue D.M., Mathisen D.J. (2009). Review of Superior Vena Cava Resection in the Management of Benign Disease and Pulmonary or Mediastinal Malignancies. Ann. Thorac. Surg..

[144] Nikolaidis E., Bolanos N., Anagnostopoulos D., Leivaditis V., Grapatsas K., Koletsis E., Papatriantafyllou A., Panagiotopoulos I., Mulita F., Baltayiannis N. (2023). Lung cancer invading the superior vena cava – surgical treatment. A short and up-to-date review. Pol. J. Cardio-Thoracic Surg..

[145] Doty D. (1982). Bypass of superior vena cava—6 years experience with spiral vein graft for obstruction of superior vena cava due to benign and malignant disease. J. Thorac Cardiovasc Surg..

[146] Siyuan L., Wada D.T., Evora P.R.B. (2023). Cardiopulmonary bypass and cancer dissemination and progression: Myth reality, enigma, puzzle?. Curr. Chall. Thorac. Surg..

[147] Li R., Luo Q., Huddleston S.J. (2024). Patients with metastatic cancer have worse short-term coronary artery bypass grafting outcomes: A population-based study of National Inpatient Sample from 2015 to 2020. Cardiovasc. Revascularization Med..

[148] Pinto C.A., Marcella S., August D., Holland B., Kostis J.B., Demissie K. (2013). Cardiopulmonary bypass has a modest association with cancer progression: A retrospective cohort study. BMC Cancer.

[149] Ryu S.W., Jeon B.B., Kim H.J., Kim J.B., Jung S.-H., Choo S.J., Chung C.H., Lee J.W. (2020). Surgical Outcomes of Malignant Primary Cardiac Tumor: A 20-Year Study at a Single Center. Korean J. Thorac. Cardiovasc. Surg..

[150] Chan J., Rosenfeldt F., Chaudhuri K., Marasco S. (2012). Cardiac Surgery in Patients with a History of Malignancy: Increased Complication Rate but Similar Mortality. Heart Lung Circ..

[151] Carrascal Y., Gualis J., Arévalo A., Fulquet E., Flórez S., Rey J., Echevarría J.R., Di Stefano S., Fiz L. (2008). Cardiac Surgery with Extracorporeal Circulation in Cancer Patients: Influence on Surgical Morbidity and Mortality, and on Survival. Rev. Esp. Cardiol..

[152] Mistiaen W.P., Van Cauwelaert P., Muylaert P., Wuyts F., Harrisson F., Bortier H. (2004). Effect of prior malignancy on survival after cardiac surgery. Ann. Thorac. Surg..

[153] Li H., Xu D., Chen Z., Ding W., Hong T., Chen H., Shao M., Lai H., Hou Y., Wang C. (2014). Prognostic Analysis for Survival After Resections of Localized Primary Cardiac Sarcomas: A Single-Institution Experience. Ann. Thorac. Surg..

[154] Khan R., Sunthankar K.I., Yasinzai A.Q.K., Tareen B., Zarak M.S., Khan J., Nasir H., Nakasaki M., Jahangir E., Heneidi S. (2023). Primary cardiac sarcoma: Demographics, genomic study correlation, and survival benefits of surgery with adjuvant therapy in U.S. population. Clin. Res. Cardiol..

[155] Sun J., Li J., Wang X., Huo X., Xu W., Li F. (2025). Primary cardiac angiosarcoma: A clinical report of 1 case and review of the literature. Medicine.

[156] Rahouma M., Mohsen H., Morsi M., Khairallah S., Azab L., Abdelhemid M., Kumar A., Ahmed M.M.E.-S. (2025). Prevalence, Diagnosis, and Treatment of Cardiac Tumors: A Narrative Review. J. Clin. Med..

[157] Jin C., Sharma A.N., Thevakumar B., Majid M., Al Chalaby S., Takahashi N., Tanious A., Arockiam A.D., Beri N., Amsterdam E.A. (2020). Carcinoid Heart Disease: Pathophysiology, Pathology, Clinical Manifestations, and Management. Cardiology.

[158] Davar J., Connolly H.M., Caplin M.E., Pavel M., Zacks J., Bhattacharyya S., Cuthbertson D.J., Dobson R., Grozinsky-Glasberg S., Steeds R.P. (2017). Diagnosing and Managing Carcinoid Heart Disease in Patients With Neuroendocrine Tumors: An Expert Statement. J. Am. Coll. Cardiol..

[159] Connolly H.M., Schaff H.V., Abel M.D., Rubin J., Askew J.W., Li Z., Inda J.J., Luis S.A., Nishimura R.A., Pellikka P.A. (2015). Early and Late Outcomes of Surgical Treatment in Carcinoid Heart Disease. JACC.

[160] Albåge A., Montibello M. (2020). Surgical aspects of valve replacement in carcinoid heart disease. J. Card. Surg..

[161] Grozinsky-Glasberg S., Davar J., Hofland J., Dobson R., Prasad V., Pascher A., Denecke T., Tesselaar M.E.T., Panzuto F., Albåge A. (2022). European Neuroendocrine Tumor Society (ENETS) 2022 Guidance Paper for Carcinoid Syndrome and Carcinoid Heart Disease. J. Neuroendocr..

[162] Nguyen A., Schaff H.V., Abel M.D., Luis S.A., Lahr B.D., Halfdanarson T.R., Connolly H.M. (2019). Improving outcome of valve replacement for carcinoid heart disease. J. Thorac. Cardiovasc. Surg..

[163] Delaney P.K., Brohan J., Bhakta P., Singh U., Williams O.E., Gormley C., Sivadasan P.C., O’BRien B. (2020). Preoperative frailty assessment predicts inferior quality of life outcomes up to one year after cardiac surgery: A prospective observational cohort study. J. Clin. Anesth..

[164] Fehlmann C.A., Bezzina K., Mazzola R., Visintini S.M., Guo M.H., Rubens F.D., Wells G.A., McGuinty C., Huang A., Khoury L. (2023). Influence of preoperative frailty on quality of life after cardiac surgery: A systematic review and meta-analysis. J. Am. Geriatr. Soc..

[165] Juliana N., Aziz N.A.S.A., Maluin S.M., Abu Yazit N.A., Azmani S., Kadiman S., Hafidz K.M., Teng N.I.M.F., Das S. (2024). Nutritional Status and Post-Cardiac Surgery Outcomes: An Updated Review with Emphasis on Cognitive Function. J. Clin. Med..

[166] Sanders J.J., Temin S., Ghoshal A., Alesi E.R., Ali Z.V., Chauhan C., Cleary J.F., Epstein A.S., Firn J.I., Jones J.A. (2024). Palliative Care for Patients with Cancer: ASCO Guideline Update. J. Clin. Oncol..

[167] Mangner N., Woitek F.J., Haussig S., Holzhey D., Stachel G., Schlotter F., Höllriegel R., Mohr F.W., Schuler G., Linke A. (2017). Impact of active cancer disease on the outcome of patients undergoing transcatheter aortic valve replacement. J. Interv. Cardiol..

[168] Lancellotti P., Suter T.M., López-Fernández T., Galderisi M., Lyon A.R., Van der Meer P., Solal A.C., Zamorano J.-L., Jerusalem G., Moonen M. (2018). Cardio-Oncology Services: Rationale, organization, and implementation. Eur. Hear. J..

